# Plk1 bound to Bub1 contributes to spindle assembly checkpoint activity during mitosis

**DOI:** 10.1038/s41598-017-09114-3

**Published:** 2017-08-18

**Authors:** Masanori Ikeda, Kozo Tanaka

**Affiliations:** Department of Molecular Oncology, Institute of Development, Aging and Cancer, Tohoku University, 4-1 Seiryo-machi, Aoba-ku, Sendai, Miyagi 980-8575 Japan

## Abstract

For faithful chromosome segregation, the formation of stable kinetochore–microtubule attachment and its monitoring by the spindle assembly checkpoint (SAC) are coordinately regulated by mechanisms that are currently ill-defined. Here, we show that polo-like kinase 1 (Plk1), which is instrumental in forming stable kinetochore–microtubule attachments, is also involved in the maintenance of SAC activity by binding to Bub1, but not by binding to CLASP2 or CLIP-170. The effect of Plk1 on the SAC was found to be mediated through phosphorylation of Mps1, an essential kinase for the SAC, as well as through phosphorylation of the MELT repeats in Knl1. Bub1 acts as a platform for assembling other SAC components on the phosphorylated MELT repeats. We propose that Bub1-bound Plk1 is important for the maintenance of SAC activity by supporting Bub1 localization to kinetochores in prometaphase, a time when the kinetochore Mps1 level is reduced, until the formation of stable kinetochore-microtubule attachment is completed. Our study reveals an intricate mechanism for coordinating the formation of stable kinetochore–microtubule attachment and SAC activity.

## Introduction

Faithful chromosome segregation during mitosis is fundamental to the maintenance of genetic information. A prerequisite for faithful chromosome segregation is the establishment of bi-orientation, which is the attachment of sister kinetochores on all replicated chromosomes to spindle microtubules from opposite spindle poles^1^. Mechanisms exist to facilitate the formation of correct attachments, as well as to correct erroneous attachments^1^. To ensure bi-orientation establishment for all the chromosomes, there is also a mechanism to restrain chromosome segregation until all the kinetochores form stable attachments to microtubules; this is called the spindle assembly checkpoint (SAC)^2^. Bi-orientation establishment and the SAC are coordinately regulated on kinetochores, mainly at the outer kinetochore. This comprises three structural complexes, collectively called the KMN (Knl1–Mis12–Ndc80) network^3^. Among these, the Ndc80 complex plays a major role in the attachment to microtubules, while the Knl1 complex functions as a platform for the assembly of the SAC machinery. Coordination between the bi-orientation establishment and the SAC depends on an elaborate protein interaction network, in which phosphorylation by mitotic kinases plays a key role^2^. Mitotic kinases (e.g., Aurora A, Aurora B, Plk1, Mps1, Bub1) function by phosphorylating specific substrates, depending on their consensus sequences and intracellular localization and are counteracted by opposing phosphatase activities. In addition, kinases often affect each other by direct phosphorylation, regulation of their localization, and recruitment of opposing phosphatases, among many other activities. The detailed picture of the interrelationship between mitotic kinases, however, remains unclear.

Plk1 (polo-like kinase 1) is one of the conserved mitotic kinases, contributing to processes such as mitotic entry, bipolar spindle formation, and kinetochore–microtubule attachment^4^. Plk1 localizes to kinetochores through interaction with a number of kinetochore proteins^5–10^. When Plk1 is depleted or inhibited, cells arrest in mitosis due to SAC activation by defective kinetochore–microtubule attachments^11–13^. The phosphorylation consensus sequence of Plk1 was reported to be similar to that of another mitotic kinase, Mps1 (monopolar spindle 1)^14^. Mps1 resides at the top of the SAC signalling pathway; it triggers the assembly of SAC components on unattached kinetochores by phosphorylating Knl1 at the MELT repeats, leading to binding of Bub1–Bub3 to the phosphorylated MELT repeats. This is followed by recruitment of other SAC components such as BubR1, Mad1, and Mad2^15–19^. Mps1 is activated by autophosphorylation^20, 21^, and activated Mps1 is highly dynamic on kinetochores^22^. Recent reports showed that Mps1 localizes to kinetochores for SAC activation by binding to the N-terminal region of Hec1, a component of the Ndc80 complex. Microtubule attachment to Hec1 results in Mps1 exclusion from kinetochores, thus linking kinetochore–microtubule attachment to SAC silencing^23, 24^.

Here, we report that Plk1, known to function in kinetochore–microtubule attachment, also works on SAC regulation. Starting from the observation that kinetochore localization of Mps1 increased in Plk1-inhibited cells, we revealed that Plk1 phosphorylates Mps1 as well as Knl1, and plays a role in maintaining SAC activity, consistent with recent reports^25, 26^. Moreover, we found that the fraction of Plk1 recruited to kinetochores by binding to Bub1 plays a major role in this function. Our results illustrate a multi-layered, bi-directional interrelationship between mitotic kinases that enables intricate regulation of the SAC.

## Results

### Plk1 phosphorylates Mps1 and affects its kinetochore localization

**Figure 1:**
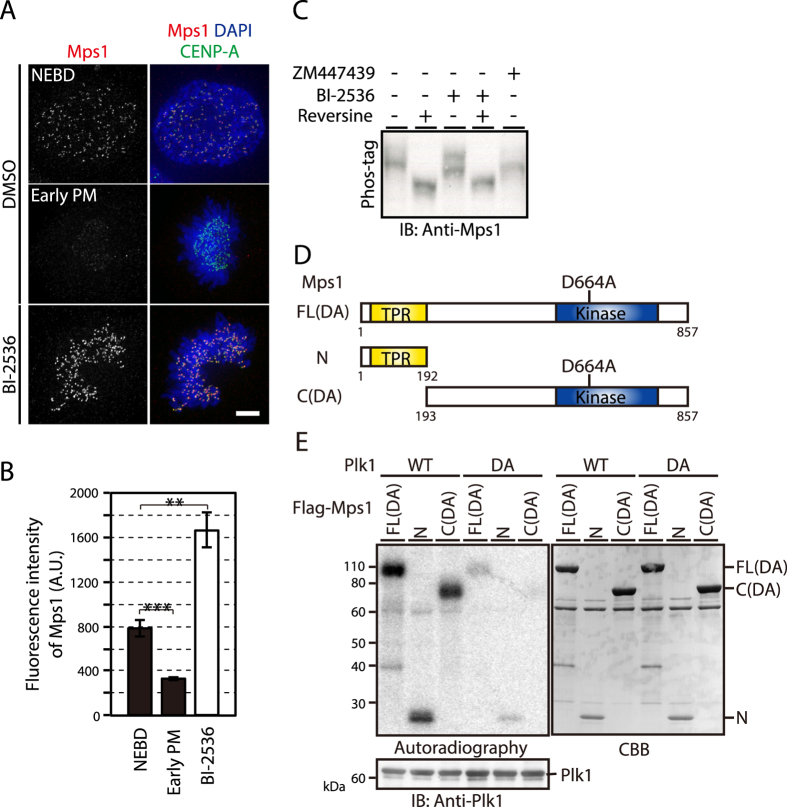
Plk1 phosphorylates Mps1 and affects its kinetochore localization. (**A**) Kinetochore localization of Mps1 in Plk1-inhibited cells. Mitotic HeLa cells expressing CENP-A-GFP (green) during nuclear envelope breakdown (NEBD) and in early prometaphase (PM), as well as in the presence of BI-2536, were fixed and immunostained with an antibody against Mps1 (red) and counterstained with 4,6-diamidino-2-phenylindole (DAPI) to label DNA (blue). Scale bar: 5 μm. (**B**) Kinetochore Mps1 levels during NEBD, in early PM, and in the presence of BI-2536 were quantified. At least 91 kinetochores per cell (5 cells) were counted for each condition. The experiments were repeated three times, and the representative data obtained from a single experiment were shown. A.U: arbitrary units. Error bars represent S.E. **P < 0.001; ***P < 0.0005 (two-tailed *t*-test). (**C**) Bandshift of Mps1 in cells treated with reversine (500 nM), BI-2536 (300 nM), or ZM447439 (2 μM). Samples were subjected to Phos-tag SDS-PAGE followed by western blotting analysis using an antibody against Mps1. (**D**) Schematic representation of Mps1 truncation mutants used in the *in vitro* kinase assay. The locations of the tetratricopeptide repeat (TPR) domain, kinase domain, and the kinase-inactivating mutation are shown. (**E**) *In vitro* kinase assay of Mps1 by Plk1. The Mps1 truncation mutants shown in (**D**) were incubated with wild-type (WT) or kinase-dead (DA) Plk1 in the presence of γ-^32^P-ATP. γ-^32^P-ATP incorporated into the Mps1 constructs was detected by autoradiography.

First, we observed kinetochore localization of Mps1 in HeLa cells in the presence of BI-2536, a potent and specific inhibitor of Plk1^12, 13^. We found that the Mps1 level at kinetochores sharply decreased in early prometaphase (PM) after nuclear envelope breakdown (NEBD; Fig. 1A,B). Intriguingly, in Plk1-inhibited cells, kinetochore localization of Mps1 significantly increased, to a level even higher than that during NEBD (Fig. 1A,B). Depletion of Plk1 in HeLa cells by RNAi in the presence of Eg5 inhibitor III, which abolishes spindle bipolarity by inhibiting Eg5 (kinesin-5) and arrests cells in PM by activating the SAC, also induced kinetochore accumulation of Mps1, confirming the effect of Plk1 inhibition on kinetochore localization of Mps1 (Supplementary Fig. S1A–C). Interestingly, the increase in Mps1 localization to kinetochores in the presence of BI-2536 was not evident when cells were treated with nocodazole, a microtubule depolymerizer (Supplementary Figs S1D, S2C,D), suggesting that Plk1 regulates Mps1 kinetochore localization through microtubules. It was reported that Mps1 directly binds to microtubules via its C-terminal region, which contains a kinase domain^27^. Therefore, we performed a microtubule-pelleting assay to test the effect of Plk1 inhibition on the binding of Mps1 to microtubules. As shown in Supplementary Fig. S1E, we found that Mps1 was co-pelleted with microtubules stabilized by Taxol. However, there was no increase in Mps1 in the co-pelleted fraction in the presence of either BI-2536 or reversine, a selective Mps1 inhibitor^28^, suggesting that Mps1 or Plk1 activity does not affect the microtubule-binding ability of Mps1. Collectively, our data indicate that Plk1 inhibition increases Mps1 localization to kinetochores, which depends on the presence of microtubules in an unidentified manner.

Next, we examined whether Plk1 inhibition by BI-2536 affects Mps1 interaction with Hec1, which targets Mps1 to kinetochores^23, 24, 29^. We expressed GFP-Mps1 in HeLa cells and detected Hec1 co-precipitated with GFP-Mps1 that was immunoprecipitated using an anti-GFP antibody. More Hec1 co-precipitated with GFP–Mps1 in the presence of reversine than in controls (Supplementary Fig. S1F,G), consistent with the notion that kinetochore localization of Mps1 is increased by Mps1 inhibition. In the presence of BI-2536, more Hec1 co-precipitated with GFP-Mps1, and the co-precipitation was increased still further in the presence of both inhibitors (Supplementary Fig. S1F,G).

Then, we asked whether Plk1 phosphorylates Mps1. First, we examined Mps1 phosphorylation in HeLa cells during mitosis in the presence of BI-2536. Retardation of the electrophoretic mobility of Mps1 in Phos-tag™ gel was abolished in the presence of reversine (Fig. 1C), as expected. In contrast, the electrophoretic mobility shift was partially ameliorated in the presence of BI-2536 (Fig. 1C), showing that Plk1 is also involved in the phosphorylation of Mps1. Co-inhibition of both Mps1 and Plk1 did not increase the bandshift further compared to Mps1 inhibition alone (Fig. 1C). Aurora B is also involved in Mps1 phosphorylation; a bandshift of Mps1 was seen in the presence of an Aurora B inhibitor, ZM447439 (Fig. 1C), as previously suggested^29^. Next, we investigated the phosphorylation of Mps1 by Plk1 *in vitro*. We purified Flag-tagged Mps1 expressed in insect cells. To inhibit autophosphorylation of Mps1, a mutation was introduced into the kinase domain (D664A) to abrogate kinase activity (Fig. 1D)^30^. We also prepared two deletion constructs of Mps1: Flag-Mps1-N (aa1–192) and Flag-Mps1-C (aa193–857, D664A). We incubated these constructs with purified Plk1 in the presence of γ-^32^P-ATP and detected phosphorylation using autoradiography (Fig. 1E). We found that both Flag-Mps1-N and Flag-Mps1-C were phosphorylated, as well as the full-length Flag-Mps1 (Fig. 1E), in agreement with a previous report^25^. The phosphorylation was dependent on Plk1, as it was not seen in the presence of kinase-dead Plk1 (D194A; Fig. 1E). We further examined whether Plk1 interacts with Mps1, because Plk1 interacts with its substrates at the docking site phosphorylated by Cdk1 through its polo-box domain (PBD)^31, 32^, and a previous report demonstrated that Cdk-dependent phosphorylation is required for potentiation of *Xenopus* Mps1 activity to sustain the checkpoint function in mitosis^33^. We expressed and purified Flag-tagged Mps1 and Plk1 from insect cells and examined whether Plk1 is co-immunoprecipitated with Flag-Mps1 in an immunoprecipitation assay using anti-Flag antibody. Mps1 expressed in insect cells was phosphorylated, which was suppressed by a Cdk1 inhibitor (data not shown). Plk1 was not co-immunoprecipitated with Mps1, irrespective of their kinase activities (Supplementary Fig. S1H). We also assessed the interaction of Mps1 with Plk1 in a GST pull-down assay. When mitotic cell lysates were incubated with full length or the N- or C-terminal fragment of GST-tagged Plk1 bound to glutathione-Sepharose beads, Mps1 was not detected with any of the GST-Plk1 constructs. As a positive control, we detected BubR1, a known Plk1-binding protein via PBD, with full length Plk1 and the C-terminal fragment containing PBD (Supplementary Fig. S1I). These results suggest that Mps1 phosphorylation by Plk1 is not through the binding of Plk1 to Mps1 via PBD. Overall, these data suggest that Plk1 phosphorylates Mps1, and Plk1 inhibition increases the localization of Mps1 to kinetochores in a microtubule-dependent manner.

### Plk1 is required for full activation of Mps1 on kinetochores to maintain SAC activity

**Figure 2:**
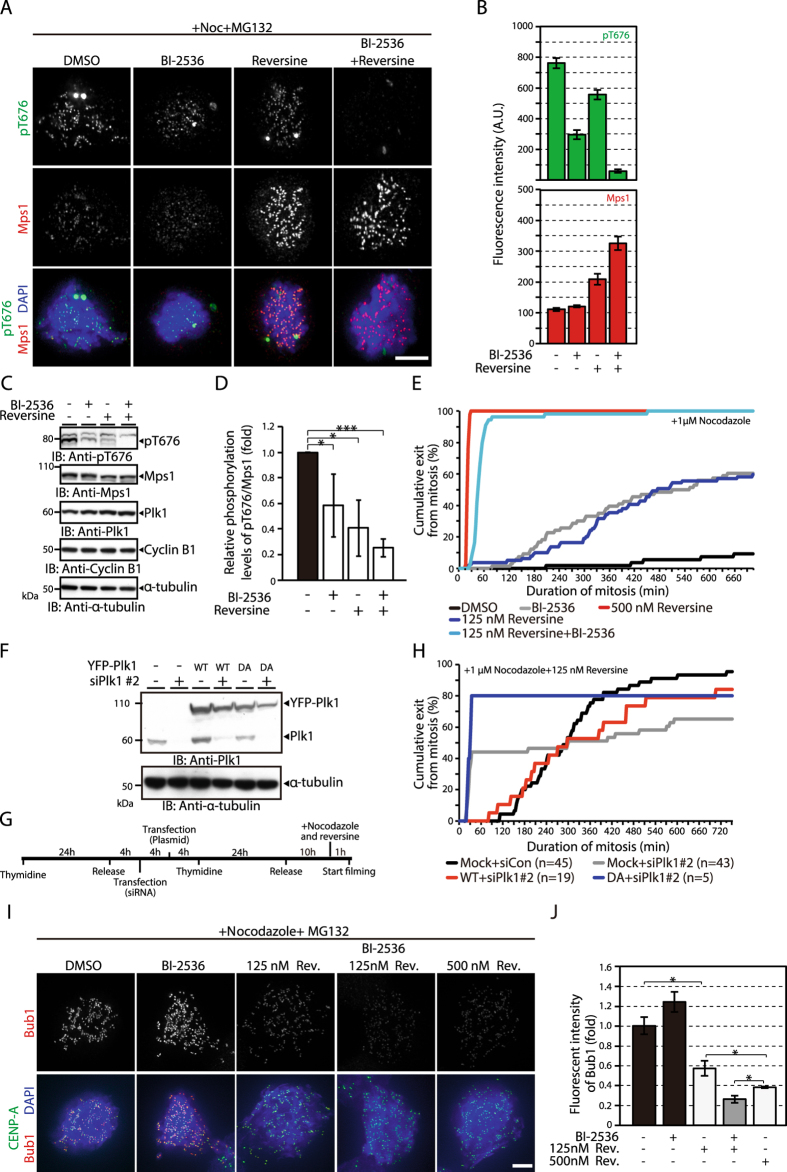
Plk1 is involved in the activation of Mps1 on kinetochores and the maintenance of SAC activity. (**A**) Phosphorylation of Mps1 on kinetochores in Plk1- and/or Mps1-inhibited HeLa cells. Mitotic HeLa cells were treated with 300 nM BI-2536 and/or 500 nM reversine in the presence of nocodazole and MG132, fixed and stained with antibodies against phospho-Thr676 (pT676) of Mps1 (green) and Mps1 (red). DNA was stained with DAPI (blue). Scale bar: 5 μm. (**B**) Quantification of pT676 and total Mps1 signal on kinetochores. The fluorescence intensity of pT676 of Mps1 and total Mps1 in cells treated as in (**A**) is shown. At least 76 kinetochore signals per cell (5 cells) were quantified for each condition. The experiments were repeated three times, and the representative dataset collected from a single experiment was shown. A.U: arbitrary units. Error bars represent S.E. (**C**) Phosphorylation of Mps1 in Plk1- and/or Mps1-inhibited cells. HeLa cells treated with 300 nM BI-2536 and/or 500 nM reversine were lysed and subjected to western blotting analysis with antibodies as indicated. (**D**) Quantification of pT676 to total Mps1 ratio in HeLa cells treated as in (**C**). The ratio in DMSO-treated cells was set as 1. Error bars represent S.D. of three experiments. *P < 0.05; *** *P* < 0.0001 (two-tailed *t*-test). (**E**) Plk1 contributes to SAC maintenance. The cumulative frequency of cells treated with reversine and/or BI-2536 that exited from mitotic arrest in the presence of nocodazole is shown. At least 43 cells were measured for each condition from a single experiment, representing three independent experiments. (**F**) Depletion of Plk1 with an siRNA targeting the untranslated region (3′-UTR) of Plk1 and the expression of YFP-Plk1. The lysates were separated by SDS-PAGE and probed by western blotting with an anti-Plk1 antibody or anti-α-tubulin antibody. (**G**) Schematic of the experimental procedure in (**F**) and (**H**). (**H**) Graph showing the cumulative frequency of cells exiting from mitosis in the presence of 1 μM nocodazole and 125 nM reversine. Cells were transfected with mock or Plk1 siRNA (3′-UTR), followed by transfection with wild-type (WT) or a kinase-dead mutant (DA) of YFP-Plk1. The indicated number of cells were analysed for each condition from a single experiment, representing three independent experiments. (**I**) Kinetochore localization of Bub1 in cells treated with reversine and/or BI-2536. Cells expressing CENP-A-GFP (green) arrested in mitosis with 1 μM nocodazole and 10 μM MG132 were incubated with reversine and BI-2536 before fixation. Immunostaining was performed with an anti-Bub1 antibody (red). DNA was stained with DAPI (blue). Scale bar: 5 μm. (**J**) Quantification of Bub1 level on kinetochores. Fluorescence intensity of Bub1 in cells treated as in (**I**) is shown. Signal intensity in DMSO-treated cells was set as 1. At least 116 kinetochores per cell (5 cells) were quantified for each condition from a single experiment, representing three independent experiments. Error bars represent S.E. * *P* < 0.05 (two-tailed *t*-test).

Because active Mps1 localization is highly dynamic, Mps1 inhibition increases the level of total Mps1, but not active Mps1, on kinetochores^34, 35^. To investigate whether Plk1 inhibition also shows the same effect, we observed the level of active Mps1, which can be detected by a phospho-specific antibody against phosphorylated threonine at aa676 of Mps1 (pT676)^36^, as well as total Mps1 on kinetochores in the presence of BI-2536. As previously shown, total Mps1 on kinetochores decreased after NEBD (Supplementary Fig. S2A,B). The level of activated Mps1 on kinetochores also decreased, but to a lesser extent (Supplementary Fig. S2A,B). Plk1 inhibition did not affect either total or activated Mps1 levels during NEBD but increased the total Mps1 level in early PM, as shown in the previous section (Supplementary Fig. S2A,B). In contrast, the activated Mps1 level was not significantly changed by Plk1 inhibition in early PM (Supplementary Fig. S2A,B). Thus, the ratio of activated Mps1 to total Mps1 on kinetochores increased in PM compared to that during NEBD, but it was significantly decreased upon Plk1 inhibition (Supplementary Fig. S2B). These data suggest that Plk1 inhibition precludes Mps1 from full activation, reducing the activated fraction while increasing the total level detected on kinetochores. Next, we verified Mps1 activity in Plk1-inhibited cells in the presence of nocodazole (Fig. 2A,B). Even though total Mps1 on kinetochores was not changed by BI-2536 treatment in the absence of microtubules, as already shown in Supplementary Fig. S1D, activated Mps1 on kinetochores significantly decreased (Fig. 2A,B). Reversine treatment increased total Mps1 but not activated Mps1 (Fig. 2A,B), as previously reported^37^. Note that cells were treated with MG132, a proteasome inhibitor, to prevent cells from exiting mitosis under reduced Mps1 activity. When cells were treated with both BI-2536 and reversine, Mps1 activity on kinetochores was completely suppressed to background levels, while total Mps1 on kinetochores increased, indicating that the activity of both kinases is required for full activation of Mps1 (Fig. 2A,B). These data are consistent with a previous report^25^. When we compared the levels of activated and total Mps1 in untreated cells and the levels in cells treated with nocodazole or Eg5 inhibitor III, the activated to total Mps1 ratio was similarly decreased by Plk1 inhibition under all of the conditions despite the difference in the respective Mps1 levels (Supplementary Fig. S2C,D). This suggests that the presence of microtubules equally affects the total and activated fractions of Mps1 on kinetochores upon Plk1 inhibition. We then assessed the level of Mps1 phosphorylation with a western blotting analysis. Plk1 inhibition reduced the level of phosphorylation at T676 as seen in Mps1 inhibition, and inhibition of both kinases further reduced the phosphorylation level (Fig. 2C,D). Overall, our results suggest that Plk1 is required for full phosphorylation and activation of Mps1, together with Mps1 itself.

Our finding that Plk1 phosphorylates and activates Mps1 suggests that Plk1 is involved in SAC activation, although it has previously been shown that Plk1 inhibition causes mitotic arrest due to defects in kinetochore–microtubule attachment^11–13^. To explore the role of Plk1 in the SAC, we arrested HeLa cells in mitosis using nocodazole treatment and observed the time to exit mitosis after Plk1 inhibition. Most of the DMSO-treated control cells stayed in mitosis during the time course (Fig. 2E). Cells treated with BI-2536 did not enter the next cell cycle but gradually died in mitosis (Fig. 2E, Supplementary Fig. S3A,B), as previously reported^12, 13^. To enable a more sensitive detection of the role of Plk1 in the SAC, we partially suppressed SAC activation using a sub-optimal dose of reversine. Cells quickly exited mitosis in the presence of 500 nM reversine, but when the reversine concentration was reduced to 125 nM, the timing of mitotic exit was substantially delayed (Fig. 2E, Supplementary Fig. S3A,B). Intriguingly, when BI-2536 was added together with 125 nM reversine, the timing of mitotic exit was significantly accelerated and was comparable to the effect of 500 nM reversine (Fig. 2E, Supplementary Fig. S3A,B). These data indicate that Plk1 contributes to SAC activation, consistent with a previous report^25^. We confirmed the finding by depleting Plk1 with RNAi (Fig. 2F). Cells were synchronized by double thymidine treatment and transfected with Plk1 siRNA between thymidine treatments (Fig. 2G). After release from the 2^nd^ thymidine treatment, cells were arrested in mitosis by nocodazole treatment and observed in the presence of 125 nM reversine. Plk1-depletion by two different siRNAs together with 125 nM reversine accelerated mitotic exit, consistent with the effect of Plk1 inhibition (Supplementary Fig. S4A). The effect was specific to Plk1 depletion, as the expression of RNAi-resistant YFP-tagged Plk1 cancelled the acceleration of mitotic exit (Fig. 2F,H). In contrast, expression of kinase-dead Plk1 did not cancel the acceleration of mitotic exit (Fig. 2F,H), demonstrating that Plk1 activity is required for the maintenance of SAC activity.

Next, we assessed the exit from mitosis of nocodazole-treated cells in the presence of Plk1 or Mps1 inhibitor by monitoring the level of cyclin B. Cyclin B levels were maintained for 4 h after nocodazole treatment in the presence of BI-2536 or 125 nM reversine but decreased in the presence of both BI-2536 and 125 nM reversine, as in the case of 500 nM reversine treatment (Supplementary Fig. S4B). We also examined the formation of the mitotic checkpoint complex (MCC), which is an effector of the SAC composed of Cdc20, Mad2, BubR1, and Bub3. When Cdc20 was immunoprecipitated in nocodazole-treated HeLa cell lysates, BubR1 and Mad2 were co-precipitated (Supplementary Fig. S4C), showing the formation of the MCC. Treatment with BI-2536 alone did not affect MCC formation, while the BubR1 and Mad2 association with Cdc20 was reduced in the presence of 125 nM reversine (Supplementary Fig. S4C). The addition of BI-2536, together with 125 nM reversine, reduced the association further, to a level comparable with 500 nM reversine treatment (Supplementary Fig. S4C). As an alternative way to define the role of Plk1 on the SAC, we examined the kinetochore localization of Bub1. Bub1 is known to act as a platform for the assembly of the SAC components on kinetochores^19^; its kinetochore localization depends on the phosphorylation of the MELT repeats on Knl1^15–17^. In the presence of 500 nM reversine, the Bub1 level on kinetochores was significantly reduced, as expected, while 125 nM reversine treatment partially reduced the Bub1 kinetochore localization (Fig. 2I,J). BI-2536 treatment alone did not reduce the Bub1 level at kinetochores, but the addition of BI-2536 together with 125 nM reversine significantly reduced the Bub1 level at kinetochores (Fig. 2I,J), consistent with a previous report^26^. Collectively, our data suggest that Plk1 acts cooperatively with Mps1 to maintain SAC activity.

### Plk1 recruitment to kinetochores by Bub1 contributes to the maintenance of SAC activity

Plk1 is recruited to kinetochores through binding to various proteins, such as Bub1, PBIP1, NudC, CLASP2, dynactin subunit p27, and CLIP-170^5–10^. We determined which protein plays a major role in recruiting Plk1 to kinetochores for the maintenance of SAC activity. First, we observed the Plk1 level on kinetochores in nocodazole-treated cells depleted of Bub1, CLASP2, or CLIP170 (Supplementary Fig. S5A–C). The Plk1 level on kinetochores was reduced by approximately half when each protein was depleted (Supplementary Fig. S5B,C). Next, we examined Mps1 signals on kinetochores in cells treated with Eg5 inhibitor III. Bub1 depletion increased the Mps1 signal (Supplementary Fig. S5D,E), similar to Plk1 inhibition (Supplementary Fig. S2C,D), while CLASP2 or CLIP-170 depletion did not (Supplementary Fig. S5D,E). These data prompted us to examine the possibility that Plk1 recruitment to kinetochores through Bub1 plays a specific role in SAC activity.

**Figure 3:**
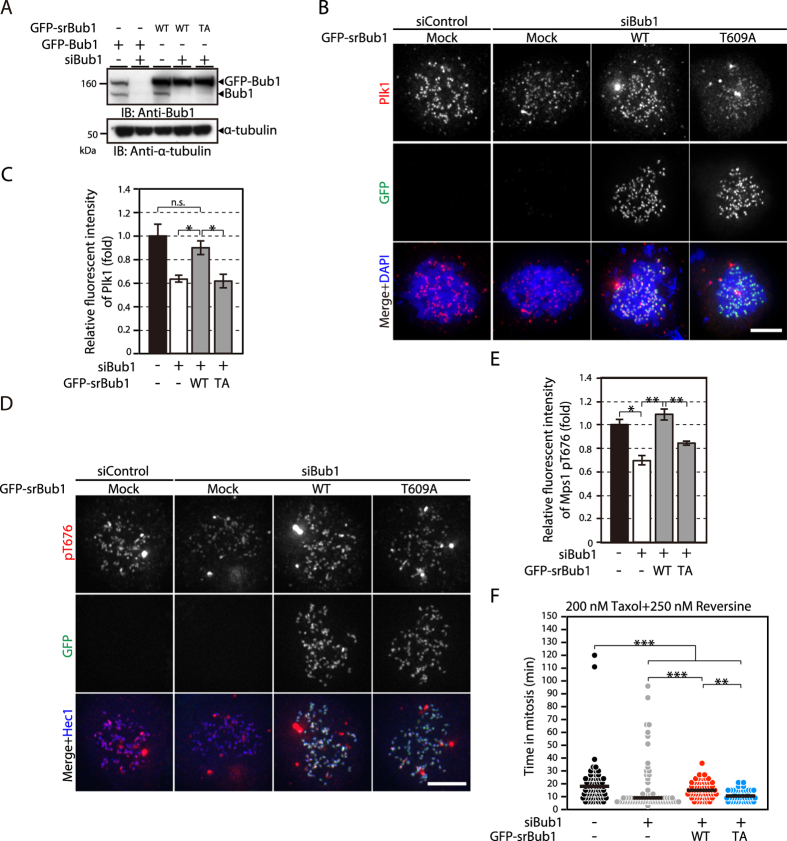
Plk1 bound to Bub1 on kinetochores promotes the kinase activity of Mps1. (**A**) Expression of RNAi-resistant GFP-Bub1 in Bub1-depleted cells. HeLa cells were transfected with mock or Bub1 siRNA, followed by transfection with GFP-Bub1 or an RNAi-resistant (sr) GFP-Bub1 construct (wild type; WT) or a mutant unable to bind to Plk1 (T609A (TA)). Cells were then lysed and subjected to western blotting analysis using antibodies against Bub1 or α-tubulin. (**B**) Kinetochore localization of Plk1 in Bub1-depleted HeLa cells expressing RNAi-resistant GFP-Bub1. HeLa cells transfected with control or Bub1 siRNA followed by transfection with a mock or RNAi-resistant GFP-Bub1 construct (wild type (WT) or T609A) were cultured for 24 h in the presence of 2 mM thymidine and released into fresh medium for 10 h. Then, the cells were incubated with 1 μM nocodazole and 10 μM MG132 for 2 h before fixation and stained with a Plk1 antibody (red) and GFP (green). DAPI was used to stain DNA (blue). Scale bar: 5 μm. (**C**) Quantification of the Plk1 signal on kinetochores. The fluorescence intensity of Plk1 in cells treated as in (**B**) was measured for at least 44 kinetochores per cell (5 cells) per condition from a single experiment, representing three independent experiments. The signal intensity in mock-treated cells was set as 1. Error bars represent S.E. *P < 0.05; n.s. *P* > 0.05 (two-tailed *t*-test) (**D**) Phosphorylation of Mps1 on kinetochores in Bub1-depleted cells. Cells were treated as in (**B**) and stained with an antibody against Mps1-pT676 (red), GFP (green), or Hec1 (blue). Scale bar: 5 μm. (**E**) Quantification of the Mps1-pT676 signal on kinetochores. The fluorescence intensity of Mps1-pT676 in cells treated as in (**D**) was measured for at least 42 kinetochores per cell (5 cells) per condition from an experiment, representing three independent experiments. The signal intensity in mock-treated cells was set as 1. Error bars represent S.E. *P < 0.05; **P < 0.001 (two-tailed *t*-test). (**F**) Mitotic duration of cells depleted of Bub1 in the presence of 200 nM Taxol and 250 nM reversine. Cells were transfected with control or Bub1 siRNA followed by transfection with a mock or RNAi-resistant GFP-Bub1 construct (WT or T609A (TA)), followed by observation of mitotic duration. More than 46 cells per condition were analysed. Dataset was obtained from a single experiment, representing three independent experiments. The median in the dot plot is indicated by a black bar. ***P < 0.0005; **P < 0.001 (Mann-Whitney *U*-test).

Bub1 recruits Plk1, in addition to other SAC components^19^, to kinetochores through binding of the PBD of Plk1 to a Cdk1-dependent phosphorylation site in Bub1^5^. It was reported that incomplete depletion of Bub1 arrests cells in mitosis, while complete depletion abolishes the SAC altogether^38^. To clarify whether, aside from its role in the recruitment of other SAC components, Bub1 recruitment of Plk1 is involved in the SAC, we expressed RNAi-resistant Bub1 constructs tagged with GFP with or without a mutation at the Plk1 binding site (T609A) in Bub1-depleted cells (Fig. 3A–C)^5^. Expression of wild-type Bub1 restored Plk1 levels on kinetochores, while Bub1-T609A did not (Fig. 3B,C), confirming the previous finding that Plk1 interacts with Bub1 through this site^5^. We then examined Mps1 activation on kinetochores in Bub1-depleted cells expressing wild-type Bub1 or Bub1-T609A (Fig. 3D,E). Expression of wild-type Bub1 restored the level of activated Mps1, phosphorylated at aa676, which was reduced by Bub1 depletion. In contrast, only a partial restoration was seen when Bub1-T609A was expressed (Fig. 3E). These data are in accordance with the idea that Plk1 recruited to kinetochores through Bub1 plays a role in SAC activity. Phosphorylation of Mps1 at aa676 was not reduced in cells depleted of CLASP2 or CLIP-170 (Supplementary Fig. S5F–I). We further verified the role of Bub1-bound Plk1 in SAC maintenance by observing mitotic exit of cells treated with microtubule inhibitors. More than half of the cells depleted of Bub1 exited mitosis in the presence of nocodazole (Supplementary Fig. S6A,B,E). In the presence of 250 nM reversine, Bub1 depletion accelerated mitotic exit (Supplementary Fig. S6A,B). These data indicate the involvement of Bub1 in the SAC. In contrast, a majority of cells depleted of CLASP2 or CLIP-170 stayed in mitosis in the presence of nocodazole, while a fraction of the cells died during mitotic arrest (Supplementary Fig. S6A,C,D,F,G). In the presence of 250 nM reversine, CLASP2 or CLIP-170 depletion did not accelerate mitotic exit (Supplementary Fig. S6A,C,D). These data show that CLASP2 and CLIP-170 are not involved in the SAC. Then, we examined the duration of mitotic arrest in cells depleted of endogenous Bub1 that instead expressed Bub1-T609A, which cannot bind to Plk1. In the presence of Taxol and 250 nM reversine, expression of wild-type Bub1, but not Bub1-T609A, restored the duration of mitosis (Fig. 3F). Altogether, our data suggest that Bub1 is primarily responsible for the role of Plk1 in SAC activity, although Plk1 recruiters other than CLASP2 and CLIP-170 may also play a role.

### Plk1 recruited to kinetochores by Bub1 promotes Knl1 phosphorylation at the MELT repeats

**Figure 4:**
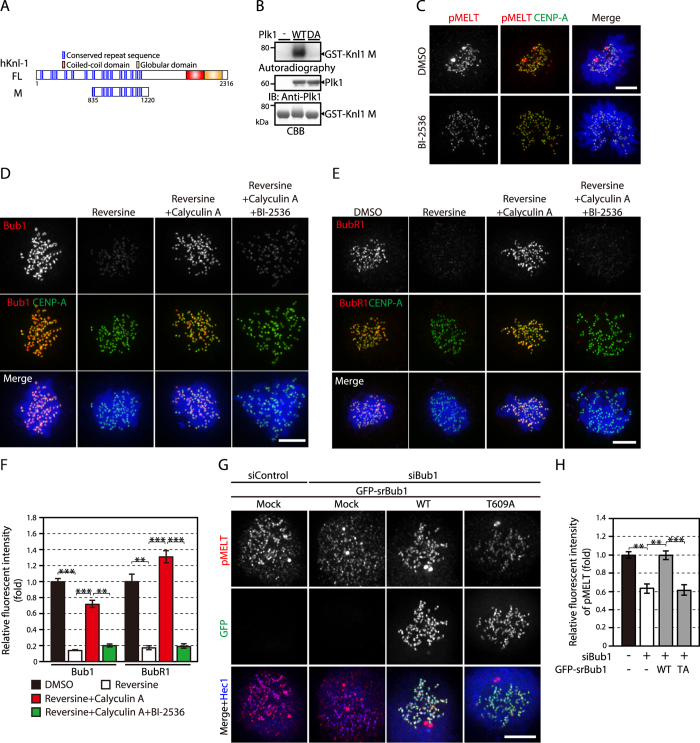
Plk1 recruited to kinetochores by Bub1 promotes Knl1 phosphorylation. (**A**) A Knl1 fragment containing the MELT repeats (hKnl1 M) is schematically shown. (**B**) Phosphorylation of the MELT repeats by Plk1. A Knl1 fragment shown in (**A**) purified from insect cells was subjected to an *in vitro* kinase assay in the presence of wild-type (WT) or kinase-dead (DA) Plk1. γ-^32^P-ATP incorporated into the Knl1 fragment was detected by autoradiography, and Plk1 was detected by western blotting. (**C**) Knl1 phosphorylation at the MELT repeats in cells treated with BI-2536. Cells expressing CENP-A-GFP (green) in early PM were treated with or without 300 nM BI-2536 and stained with an antibody against a phosphorylated MELT repeat (pMELT; red). DNA was stained with DAPI (blue). Scale bar: 5 μm. (**D**) Kinetochore localization of Bub1 in nocodazole-treated HeLa cells in the presence of 500 nM reversine with or without calyculin A and BI-2536. Cells expressing CENP-A-GFP (green) were immunostained with an antibody against Bub1 (red). DNA was stained with DAPI (blue). Scale bar: 5 μm. (**E**) Kinetochore localization of BubR1 in cells treated as in (**D**). Cells expressing CENP-A-GFP (green) were immunostained with an antibody against BubR1 (red). DNA was stained with DAPI (blue). Scale bar: 5 μm. (**F**) Quantification of Bub1 and BubR1 on kinetochores. The fluorescence intensity of Bub1 and BubR1 in cells treated as in (**D**) and (**E**) is shown. The signal intensity in DMSO-treated cells was set as 1. The intensity of Bub1 or BubR1 obtained from a single experiment, representing three independent experiments, was measured for at least 95 kinetochores per cell (5 cells) per condition. Error bars represent S.E. **P < 0.001; ***P < 0.0005 (two-tailed *t*-test). (**G**) Knl1 phosphorylation at the MELT repeats in Bub1-depleted cells. Cells were treated as in Fig. 3B and stained with an antibody against a phosphorylated MELT repeat (pMELT; red), GFP (green), or Hec1 (blue). Scale bar: 5 μm. (**H**) Quantification of Knl1-pMELT signal on kinetochores. The fluorescence intensity of Knl1-pMELT in cells treated as in (**G**) was measured for at least 42 kinetochores per cell (5 cells) per condition. Data was obtained from a single experiment, representing three independent experiments. Error bars represent S.E. **P < 0.005; ***P < 0.0005 (two-tailed *t*-test).

Bub1 is recruited to kinetochores through phosphorylation of the MELT repeats in Knl1^15–17^. The role of Plk1 on the Bub1 kinetochore localization (Fig. 2I) is thought to be indirect, through phosphorylation of Mps1 by Plk1, but the consensus phosphorylation motif shared by Mps1 and Plk1 implies that Plk1 may directly phosphorylate the MELT repeats. To verify this possibility, we first examined the phosphorylation of the MELT repeats by Plk1. A purified fragment of Knl1 spanning the middle region containing the MELT repeats (GST-Knl1 M; aa835–1220) was phosphorylated by Plk1 *in vitro*, but not by kinase-dead Plk1 (Fig. 4A,B), supporting the notion that Plk1 can directly phosphorylate the MELT repeats. In mitotic cells, phosphorylation of the MELT repeats, visualized by anti-phospho antibody against T875 of Knl1 (pMELT)^17^, was reduced by inhibiting Plk1 with BI-2536 (Fig. 4C), showing the role of Plk1 in the phosphorylation of the MELT repeats. These findings are in agreement with a recent report^25^. It was shown that phosphorylation of the MELT repeats is counteracted by phosphatase activity, regulating the localization of Bub1 and other SAC components^39^. Therefore, we hypothesized that phosphorylation of the MELT repeats is retained by Plk1 even if Mps1 activity is inhibited when the phosphatase activity is suppressed. To address this possibility, we examined recruitment of Bub1 to kinetochores in cells treated with reversine as well as calyculin A, a phosphatase inhibitor selective for PP1 and PP2A^40^. In these cells, Bub1 level at kinetochores was significantly higher compared to that in cells treated with reversine alone (Fig. 4D,F). Intriguingly, when Plk1 was inhibited together with Mps1 and phosphatases, the Bub1 level declined (Fig. 4D,F). We also examined the kinetochore localization of BubR1, which localizes to kinetochores through binding to Bub1^41^ or directly to the phosphorylated MELT repeats^42^. Again, phosphatase inhibition increased the BubR1 level in Mps1-inhibited cells, and this was reduced by Plk1 inhibition (Fig. 4E,F). These results suggest that Plk1 can promote kinetochore recruitment of Bub1 and BubR1 through direct phosphorylation of the MELT repeats.

Next, we addressed whether Plk1 bound to Bub1 phosphorylates the MELT repeats. Phosphorylation of the MELT repeats was significantly decreased by Bub1 depletion and recovered by the expression of wild-type Bub1 tagged with GFP (Fig. 4G,H). Notably, expression of Bub1–T609A did not restore phosphorylation of the MELT repeats (Fig. 4G,H), indicating that recruitment of Plk1 to kinetochores by Bub1 promotes phosphorylation of the MELT repeats. In contrast, phosphorylation of the MELT repeats did not change by depletion of CLASP2 or CLIP-170, which is not involved in the SAC (Supplementary Fig. S5J–M). In addition, we investigated whether reduced phosphorylation of the MELT repeats in Bub1-T609A-expressing cells is counteracted by phosphatase inhibition. Expression of GFP-Bub1-T609A in Bub1-depleted cells exposed to cantharidic acid or tautomycin, potent and selective inhibitors for PP1 and PP2A^43, 44^, restored phosphorylation of the MELT repeats (Supplementary Fig. S7A,B), indicating that Plk1 bound to Bub1 at kinetochores counteracts phosphatase activity to maintain phosphorylation level of the MELT repeats.

### Plk1 recruited to kinetochores by artificially tethered Bub1 augments Knl1 phosphorylation in prometaphase

**Figure 5:**
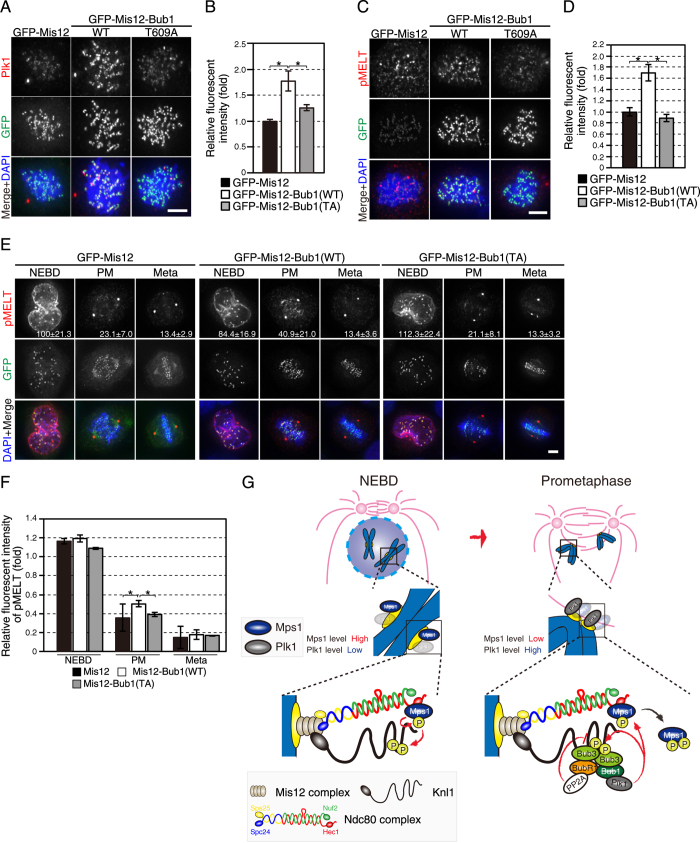
Bub1 artificially anchored to kinetochores augments Knl1 phosphorylation during prometaphase by recruiting Plk1. (**A**) Plk1 signal on kinetochores in cells expressing GFP–Mis12-Bub1. Cells transfected with a GFP-tagged Mis12 or Mis12–Bub1 construct (wild type (WT) or T609A) were arrested in mitosis by nocodazole treatment, fixed and stained with a Plk1 antibody (red) or GFP (green). DNA was labelled with DAPI (blue). Scale bar: 5 μm. (**B**) Quantification of the Plk1 signal on kinetochores. The signal intensity of Plk1 on kinetochores in cells treated as in (**A**) was measured for at least 51 kinetochores per cell (5 cells) per condition. The experiments were repeated three times, and the representative data obtained from a single experiment were shown. Error bars represent S.E. *P < 0.05 (two-tailed *t*-test). (**C**) Knl1 phosphorylation at the MELT repeats in HeLa cells expressing GFP-Mis12–Bub1. Cells were treated as in (**A**) and stained with an antibody against phosphorylated MELT repeats (pMELT; red) or GFP (green). DNA was counterstained with DAPI (blue). Scale bar: 5 μm. (**D**) Quantification of Knl1-pMELT signal on kinetochores. The fluorescence intensity of Knl1-pMELT in cells treated as in (**C**) was quantified for at least 31 kinetochores per cell (5 cells) per condition from a single experiment, representing three independent experiments. Error bars represent S.E. *P < 0.05 (two-tailed *t*-test). (**E**) Knl1 phosphorylation at the MELT repeats in early mitosis (NEBD, prometaphase (PM), metaphase (Meta)) in cells expressing GFP-Mis12–Bub1. Cells transfected with GFP-tagged Mis12 or Mis12–Bub1 construct (wild type (WT) or T609A) were released from thymidine block for 10 h, fixed and stained with an antibody against a phosphorylated MELT repeat (pMELT; red) or GFP (green). DAPI was used to counterstain DNA (blue). Scale bar: 5 μm. (**F**) Quantification of the Knl1-pMELT signal on kinetochores. The fluorescence intensity of Knl1-pMELT in cells treated as in (**E**) was measured for at least 40 kinetochores per cell (5 cells) per condition. The experiments were repeated three times, and the representative data obtained from a single experiment were shown. The signal intensity in GFP-Mis12-expressing cells during NEBD was set as 1. Error bars represent S.E. *P < 0.05 (two-tailed *t*-test). (**G**) A model depicting the regulation of SAC activity by Plk1 bound to Bub1. Just before NEBD, the maximum amount of Mps1 localizes on kinetochores and phosphorylates multiple MELT motifs in Knl1 to activate the SAC, leading to the recruitment of SAC components such as Bub3, Bub1 and BubR1. Mps1 is activated by phosphorylation at multiple sites either by Mps1 itself or Plk1, and kinetochore Mps1 sharply decreases in PM. In contrast, Plk1 at kinetochores is elevated in PM through binding to Bub1 phosphorylated by Cdk1. Plk1 bound to Bub1 plays a role in the maintenance of SAC activity in PM, counteracting phosphatases such as PP2A-B56.

We wished to explore further the role of Bub1 as a Plk1 recruiter to kinetochores in maintaining SAC activity. We artificially tethered Bub1 to kinetochores as a fusion protein with the outer kinetochore component Mis12 and observed Knl1 phosphorylation. Plk1 levels at kinetochores increased in nocodazole-treated HeLa cells expressing GFP-Mis12–Bub1-WT, but not in cells expressing GFP-Mis12 or GFP-Mis12–Bub1-T609A (Fig. 5A,B), as expected. Accordingly, phosphorylation of the MELT repeats significantly increased in cells expressing GFP-Mis12–Bub1-WT, compared with cells expressing GFP-Mis12 or GFP-Mis12–Bub1-T609A (Fig. 5C,D). A similar effect was also observed when Plk1 was directly tethered to Mis12, but not when Plk1 was tethered to Hec1, localizing to an outer position in kinetochore than Mis12 (Supplementary Fig. S8A–D), suggesting that positions of Plk1 recruiters in kinetochore determine the efficiency of phosphorylation. To further verify this notion, we examined whether Plk1 bound to Bub1 contributes to the phosphorylation of the KARD domain (pKARD) of BubR1, a known Plk1 substrate which promotes the interaction of BubR1 to PP2A^45^. We found that Bub1–T609A expression restored pKARD to the similar level achieved by the expression of wild-type Bub1 (Supplementary Fig. S8E,F), suggesting that Plk1 bound to Bub1 does not play a major role in the phosphorylation of the KARD domain. Insufficient rescue of pKARD by the expression of wild-type Bub1 may be due to the overexpression of GFP–Bub1 that resulted in the reduced fraction of BubR1 bound to GFP–Bub1 at kinetochores. Phosphorylation of the KARD domain was not markedly reduced in cells depleted of CLASP2 and/or CLIP-170 (Supplementary Fig. S8G,H). These data suggest that phosphorylation of the KARD domain by Plk1 is mainly carried out by recruiters other than Bub1, CLASP2, and CLIP-170.

Mps1 accumulates on kinetochores just before NEBD and rapidly decreases after NEBD (Fig. 1A,B). In contrast, kinetochore Plk1 increases after NEBD, reaching a maximum level in PM^10^. Therefore, one idea is that Plk1 bound to Bub1 contributes to phosphorylation of the MELT repeats after reduction of Mps1 at kinetochores in PM. Quantitative analysis showed that phosphorylation level of the MELT repeats was highest at NEBD, declined in PM, and disappeared to background level in metaphase in GFP-Mis12-expressing cells (Fig. 5E). Notably, levels of MELT phosphorylation in GFP-Mis12–Bub1-WT-expressing cells increased only in PM, compared with cells expressing GFP-Mis12. This increment was not observed in cells expressing GFP-Mis12–Bub1-T609A, suggesting that enforced recruitment of Plk1 to kinetochores by artificially tethered Bub1 augments Knl1 phosphorylation in PM, in accordance with the idea that Plk1 bound to Bub1 supports phosphorylation of the MELT repeats in PM when the kinetochore Mps1 level is reduced.

## Discussion

In this study, we addressed the role of Plk1 in the SAC; Plk1 phosphorylates Mps1 and the MELT repeats of Knl1, regulates kinetochore localization of Mps1 and Bub1, and thus contributes to SAC activity. Our results are in agreement with previous reports^4, 26^. We found that both N- and C-terminal fragments of Mps1 were phosphorylated by Plk1 (Fig. 1E), in accordance with the findings that Plk1 phosphorylation sites are distributed throughout the Mps1 sequence^4, 14^. Active Mps1 is highly dynamic on kinetochores, and the increased kinetochore localization of Mps1 in Plk1-inhibited cells (Fig. 1A) was revealed to be through the inhibition of Mps1 activity (Supplementary Fig. S2A,B). One intriguing finding was that increased kinetochore localization of Mps1 by Plk1 inhibition depends on microtubules (Supplementary Fig. S1D). In contrast, increased kinetochore localization of Mps1 as a result of its own inhibition by reversine was not affected by microtubules (Fig. 2A,B). The results of the microtubule-pelleting assay suggest that microtubule-binding activity of Mps1 is independent of its phosphorylation by Mps1 and Plk1 (Supplementary Fig. S1E). It was recently suggested that ARHGEF17 bound to Mps1 is involved in the rapid turnover of Mps1 on kinetochores^46^; it would be interesting to see how ARFGEF17 is related to the regulation of Mps1 localization by microtubules.

Although the contribution of Plk1 to SAC was typically shown in *C. elegans*, in which the intrinsic absence of Mps1 is compensated by Plk1^26^, the role of Plk1 in the SAC in mammalian cells is largely dispensable and becomes apparent only when Mps1 is partially suppressed (Fig. 2E,H) or when phosphatases are inhibited (Fig. 4D–F). While Mps1 localization to kinetochores significantly decreases upon NEBD (Fig. 1A), the Plk1 level on kinetochores is greatest in PM and a reduced amount remains in metaphase^10^. We found that Plk1 recruited to kinetochores by artificially tethering Bub1 augmented Knl1 phosphorylation at the MELT repeats in PM, but not during NEBD or metaphase (Fig. 5E,F). This suggests the possibility that Plk1 is required to maintain full SAC activity in PM when the majority of Mps1 leaves kinetochores, counteracting the opposing phosphatase activity (Fig. 5G).

We have found that Plk1 recruited to kinetochores by Bub1, but not by CLASP2 or CLIP-170, mainly contributes to the SAC, suggesting differential roles of Plk1 depending on its binding proteins. The lack of effect of Plk1 bound to Bub1 on phosphorylation of the KARD domain of BubR1 (Supplementary Fig. S8E,F) also supports this notion. Plk1 in distinct positions within kinetochore may phosphorylate different pools of substrates, which was suggested by a recent study^47^. Bub1 provides a platform for other SAC components and thus presumably contributes to the SAC via several distinct pathways. First, by recruiting Mad1, Mad2, BubR1, Bub3, and Cdc20, Bub1 promotes the formation of the MCC that inhibits the anaphase-promoting complex/cyclosome (APC/C), an E3 ubiquitin ligase essential for sister chromatid separation and mitotic exit^19^. Second, it was recently proposed that Bub1 acts as a scaffold to promote Cdc20 phosphorylation by Plk1 bound to Bub1, and by Bub1 itself^48^. Cdc20 is a cofactor for APC/C as well as an MCC component, and its phosphorylation was found to inhibit APC/C activation^48^. Third, Plk1 bound to Bub1 phosphorylates Mps1 and the MELT repeats of Knl1, promoting SAC activation (this study). The latter two pathways both depend on Plk1 bound to Bub1 and thus may act in parallel.

Our results illustrate the intricate regulation and coordination of kinetochore–microtubule attachment and the SAC. Plk1 is known to expedite kinetochore attachment to microtubules by recruiting PP2A to BubR1 through phosphorylation of the KARD domain, counteracting the Aurora B activity that destabilizes kinetochore–microtubule interaction^45^. Our results, and other recent reports, now revealed that Plk1 also plays a role in SAC regulation^4, 26^. The role of Plk1 in recruiting SAC components to kinetochores was also shown in *Xenopus* egg extracts^49^. Similarly, to Plk1, many SAC components and mitotic kinases are known to function both in kinetochore–microtubule attachment and the SAC^37, 45, 50–52^. The dual functions of these proteins are further coordinated by their interrelationships. Plk1 bound to Bub1 phosphorylates Mps1 and the MELT repeats of Knl1, promoting its own recruitment to kinetochores through the interaction of Bub1 with the phosphorylated MELT repeats (Fig. 5G). At the same time, Bub1 recruits BubR1 to kinetochores, and PP2A bound to BubR1 through its phosphorylation by Plk1 counteracts the phosphorylation of the MELT repeats^39^. Therefore, Plk1 promotes kinetochore–microtubule attachment and maintains the SAC on the one hand, but on the other hand, it prepares for silencing the SAC once the attachment is established. In a recent study using U2OS cells, Plk1 was reported to work on SAC maintenance together with Aurora B, by regulating each other^53^, adding another layer of regulation.

Mitotic fidelity is supposed to be ensured by the balance between these cooperating and counteracting mechanisms. Subtle changes in the balance may cause defects that are not essential for cell viability but give rise to chromosomal instability, which is related to formation and progression of cancer. Plk1 is known to be overexpressed in many cancers and is now a target for cancer therapy^54^. Further understanding of the role of Plk1 in the SAC is required to evaluate the efficacy of cancer therapy targeting this kinase.

## Materials and Methods

### Plasmid construction

cDNA coding for human Plk1 was cloned as previously described^55^. The cDNAs for human Mps1 and Bub1 were PCR-amplified from the MegaMan Human Transcriptome cDNA Library (Agilent Technologies). The cDNA coding for human Mis12 was a kind gift from Dr. T. Hirota^56^. These cDNAs were subcloned into pEGFPC1 or pEYFPC1 (Clontech). GFP-Mis12–Bub1 was created by insertion of Mis12 between GFP and Bub1 using an *Xho*I site. Similarly, GFP-Mis12-Plk1 was constructed by inserting Mis12 between GFP and Plk1. Hec1-mCherry-Plk1 constructs were gifts from Michael Lampson (Addgene plasmids #45205, 45206). The cDNAs for point mutation were generated using a QuickChange site-directed mutagenesis kit (Stratagene), according to the manufacturer’s instructions. To construct plasmids for siRNA-resistant Bub1, (sr-Bub1)-targeting sequences were altered by PCR-based mutation using the following primer: sr-Bub1 (5′-GAg TGc AAa CGa TCt CGA-3′). The cDNA coding for human Knl1 was a kind gift from Dr. M. Lampson^57^. For protein expression using a baculovirus expression system, cDNAs coding for Knl1, Plk1 and Mps1 fragments were constructed using PCR-based amplification and subcloned into pFastBac6P3 or pFastBac6P3-Flag vectors, constructed as previously described, to generate GST- or GST-Flag-tagged proteins^58^.

### Cell culture, synchronization, drug treatment and transfection

HeLa Kyoto cells, which has been tested negative for mycoplasma contamination, were cultured in DMEM supplemented with 10% foetal bovine serum (FBS). To synchronize cells in the early mitotic phase, HeLa Kyoto cells were cultured in the presence of 2 mM thymidine for 24 h, released from the thymidine block for 10 h, and then fixed and stained. For Plk1 inhibition at early mitosis, cells were treated with 300 nM BI-2536 for 2 h before fixation. To arrest cells at mitosis, 1 μM nocodazole or 1 μM Eg5 inhibitor III were added 2 h before fixation. For inhibition of kinase activity of Plk1 and/or Mps1, cells released from the thymidine block were incubated in medium containing 300 nM BI-2536 and/or the indicated concentrations of reversine with 10 μM MG132 in the presence or absence of 1 μM nocodazole for 2 h. For suppression of Plk1 activity in the absence of Mps1 and phosphatase activity, cells released from the thymidine block were treated with 1 μM nocodazole for 1 h and then treated with 300 nM BI-2536 together with 500 nM reversine and 10 nM calyculin A in the presence of 1 μM nocodazole and 10 μM MG132 for 30 min before fixation. For phosphatase inhibition, cells arrested in mitosis with 1 μM nocodazole were treated with 50 μM cantharidic acid or 3 μM tautomycin in the presence of 10 μM MG132 for 30 min. To investigate the effect of Plk1 inhibition on the SAC activity by immunoblotting, mitotic HeLa cells arrested by 0.3 μM nocodazole were selectively collected by mechanical shake-off, as previously described^55^, replated on poly-L-lysine-coated dishes, and then incubated with the indicated kinase inhibitors in the presence of 1 μM nocodazole for 0–240 min. Transfection of expression plasmids and siRNA oligonucleotides was performed using FuGENE HD Transfection reagent (Promega) and Lipofectamine RNAi MAX reagent (Invitrogen), respectively, according to the manufacturer’s instructions. Unless mentioned in the text, 100 nM siRNAs were used in each experiment. For rescue experiments in Plk1-depleted HeLa cells, Plk1 was depleted by transfection with siRNA targeting the untranslated region (3′-UTR) of Plk1 for 4 h after release from the 1^st^ thymidine block. After incubation in culture medium for 4 h, Plk1-depleted cells were transfected with wild-type or a kinase-dead mutant of Plk1 conjugated with YFP and incubated in culture medium for 4 h, followed by a 2^nd^ thymidine block for 24 h. For replacement of endogenous Bub1 with GFP-sr-Bub1, HeLa cells transfected with siRNA against Bub1 were transfected with GFP-sr-Bub1 mutants and treated with 2 mM thymidine for 24 h. After release from the thymidine block for 8–10 h, these cells were treated with the indicated drugs and then fixed and stained.

### RNAi

Synthetic oligonucleotides targeting human Plk1, Bub1 and CLIP-170 for RNAi were obtained from JBioS. The sequences were as follows: Plk1#1 (5′-CGAGCUGCUUAAUGACGAGUU-3′)^59^, Plk1#2 (5′-UUCCUUGGCUUUAUGCACAUU-3′) (3′-UTR)^60^, Bub1 (used in main figures) (5′-GAAUGUAAGCGUUCACGAA-3′)^61^, CLIP170#1 (5′-GCACAGCUCUGAAGACACCTT-3′), and CLIP170#2 (5′-CUGCAAUGACGACGAAACCTT-3′)^62^. The siRNA sequences used in the supplementary figures for targeting Bub1 and CLASP2 were obtained from Invitrogen (Stealth). The sequences were as follows: Bub1#1 (5′-UUUCCAUGUUCACUGGUGUCUGCUG-3′), Bub1#2 (5-UUUGAAGGAAGUCUGCUGACAGAGC-3), CLASP2#1 (5′-AAAUAAUGAAAUCAGCACUGACUGU-3′), and CLASP2#2 (5′-AAUUGAAGUGGCCAACACUGAUGCC-3′).

### Reagents and antibodies

Nocodazole, MG132 (Sigma), Taxol (Wako), Eg5 inhibitor III, reversine, BI-2536 (Axon Medchem), ZM447439 (Tocris Bioscience), calyculin A (Cell Signaling), cantharidic acid (Abcam) and tautomycin (Enzo Life Sciences) were commercially purchased. Phospho-antibodies against T676 of Mps1 (pT676)^36^, T680 of BubR1 (pT680)^45^ and T875 of Knl1 (pT875)^17^ were a kind gift from Dr. G.J. Kops and Dr. Y. Watanabe, respectively, and used at the following dilutions for immunoblotting (IB) and immunofluorescence (IF): Mps1 pT676 (IB 1/2000, IF 1/2000), BubR1 pT680 (IF 1/2000), Knl1 pT875 (IF 1/500). The following antibodies were commercially purchased and used at the indicated dilution: α-tubulin (B-5-1-2, T6074, Sigma, IB 1/1000, IF 1/1000), Bub1 (BETHYL, A300-373A, IB 1/1000, IF 1/1000), BubR1 (BETHYL, A300-386A, IB 1/1000, IF 1/500), Cdc20 (Protein Tech Group, 10252-1-AP, IP 1/500, IB 1/500), CLASP2 (Santa Cruz Biotechnology, sc-98440, IB 1/500, IF 1/250), CLIP170 (H-300, Santa Cruz Biotechnology, sc-25613, IB 1/500, IF 1/500), Cyclin B1 (BD Transduction Laboratories, 554177, IB 1/1000), Flag (M2, Sigma, F3165, IB 1/1000), GFP (Molecular Probes, A6455, IP 1000, IB 1/1000), Hec1 (9G3, Abcam, ab3613, IB 1/500, IF 1/500), Plk1 (F-8, Santa Cruz Biotechnology, sc-17783, IB 1/1000, IF 1/500), Mad2 (Novus Biologicals, NB100-563, IB 1/500), Mps1 (NT, clone 3-472-1, Millipore, 05-682, IB 1/250, IF; 1/1000).

### Immunostaining, fluorescence microscopy and live cell imaging

HeLa cells grown on coverslips were pre-extracted with 0.1% Triton X-100 in PHEM buffer pH 7.0 containing 60 mM PIPES, 25 mM HEPES, 10 mM EGTA and 2 mM MgSO_4_ and fixed with 3.6% formaldehyde in the same buffer at 37 °C for 10 min. For staining Mps1 and Mps1 pT676, cells were fixed with 3.6% formaldehyde in PHEM buffer containing 0.2% Triton X-100 after pre-extraction with the same buffer. Coverslips were blocked with 3% BSA in PBS containing 0.01% Triton X-100 (wash buffer) at room temperature (RT) for 30 min, and incubated with primary antibodies overnight at 4 °C and then secondary antibodies at RT for 1 h. Goat anti-rabbit IgG Alexa-Fluor 488 and 568, goat anti-mouse Alexa-Fluor 488 and 568 (Molecular Probes) were used as secondary antibodies. Fluorescence images were acquired using a Personal DV microscope (Applied Precision) equipped with a Plan Apochromat 100x oil objective lens (NA 1.40) (Olympus) mounted on an inverted microscope (CoolSNAP HQ; Photometrics), driven by softWoRx software (Applied Precision, LLC). A series of Z-stacking images acquired at 0.2 or 0.3 μm intervals were deconvoluted using an algorithm with preserved settings and presented as maximum intensity projections. For time-lapse imaging, cells were cultured in an 8-well chamber (Thermo Fisher Scientific). After thymidine block, cells were cultured for 8–9 h in Leibovitz’s L-15 medium containing 20 mM HEPES pH 7.5 and 10% FBS, and then treated with various concentration of reversine together with 1 μM nocodazole or 200 nM Taxol for at least 30 min before recording. Time-lapse imaging was performed with a Personal DV microscope, and bright field and/or H2B-mCherry images were taken using 20x dry objective lens (NA 0.75) (Olympus). Images taken at 3 min intervals were processed using ImageJ software, and graphs were produced using Microsoft Excel.

### Quantification of fluorescence intensity

Quantification of the signal intensity at kinetochores was conducted using ImageJ. To determine the position of kinetochores, the circular region encompassing CENP-A signals (Figs 1B, 2J and 4F, Supplementary Figs S1C, S5C,E,G,I,K,M and S8H), Mps1 or pT676 signals (Fig. 2B, Supplementary Fig. S2B,D) in each z section was defined as the region of interest (ROI). The fluorescence intensity of target proteins within the same ROI was measured, and background intensity was subtracted. For rescue experiments in Bub1-depleted cells, kinetochore positions were determined using Hec1 signals labelled with anti-Hec1 antibody as mentioned above (Figs 3C,E and 4H; Supplementary Figs S7B, S8F). Similarly, GFP signals on kinetochores were used to decide kinetochore positions in cells expressing GFP-Mis12 or GFP-Mis12-Bub1 (Fig. 5B,D,F). In both cases, the fluorescence intensity of target proteins per condition was measured among cells possessing a similar expression level of GFP-tagged proteins. The indicated number of kinetochores in each figure was measured and then averaged per cell. The data are presented as the mean ± S.E., and *P* values were calculated using paired two-tailed Student’s *t*-test with Microsoft Excel software. A value of *P* < 0.05 was considered to be statistically significant.

### Immunoprecipitation

For immunoprecipitation analysis, mitotic HeLa cells released from the thymidine block were arrested in early mitotic phase by 0.3 μM nocodazole for 16 h and mechanically collected as described above. Cells were lysed and sonicated in lysis buffer (20 mM Tris-HCl [pH 7.5], 150 mM NaCl, 0.1% NP-40, 10% glycerol, 1 mM EGTA, and 1 mM DTT) supplemented with phosphatase inhibitor cocktail (Nacalai) and protease inhibitor cocktail (Roche). Cell lysates were clarified by centrifugation for 10 min at 4 °C at 16,000 × *g*. Supernatants were pre-incubated with IgG-conjugated magnetic beads (Dynabeads M-280, Thermo-Fisher Scientific) for 1 h at 4 °C, incubated with anti-rabbit IgG or indicated antibodies for 1 h at 4 °C, and then incubated with the magnetic beads for 2 h at 4 °C. Precipitates were washed twice with lysis buffer containing 500 mM NaCl and three times with the same buffer containing 150 mM NaCl, and the samples were then subjected to immunoblot analysis.

### Protein purification

GST- and GST-Flag-tagged proteins were expressed in Sf-21 cells using a baculovirus expression system (Life Technologies) according to the manufacturer’s instructions. The GST-Knl1 fragment was purified using glutathione-Sepharose 4B (GE Healthcare) as previously described^55^. Un-labelled Plk1 or Flag-tagged Mps1 were purified by cleavage of GST from GST-Plk1 or GST-Flag-Mps1 using PreScission protease (GE Healthcare) in cleavage buffer containing 50 mM Tris-HCl pH 7.5, 150 mM NaCl, 0.1% NP40 and 1 mM DTT.

### GST pull-down assay

Full length or the N- or C-terminal fragment of Plk1 tagged with GST were expressed in Sf-21 cells by baculovirus and purified with glutathione-Sepharose 4B. Cell lysates were pre-incubated with protein A-Sepharose (GE Healthcare), and supernatants were incubated with GST-tagged proteins bound to glutathione-Sepharose beads. After washing twice with lysis buffer containing 500 mM NaCl and three times with the same buffer containing 150 mM NaCl, precipitates were analysed by immunoblotting.

### Kinase assay

Wild-type or kinase-dead Plk1 purified from Sf-21 cells (250 ng) were mixed with 2 μg of a kinase-dead form of Flag-tagged full length, or a fragment of Mps1 in 20 μl of kinase buffer (20 mM Tris-HCl pH 7.5, 10 mM MgCl_2_, 1 mM DTT) containing 1 mM ATP and 2.5 μCi [γ^32^P]ATP and incubated for 1 h at 30 °C. Similarly, to examine phosphorylation of Knl1 by Plk1, 2 μg of purified GST-Knl1 fragment was incubated with 250 ng of Plk1 in kinase buffer in the presence of 2.5 μCi [γ^32^P]ATP for 45 min at 30 °C. The reaction mixture was separated by SDS-PAGE and analysed by autoradiography to detect ^32^P-labelled proteins.

### Microtubule-pelleting assay

Mitotic HeLa cells treated with nocodazole after release from the thymidine block were selectively collected and re-plated on poly-L-lysine-coated dishes as described above, incubated with Plk1 and/or Mps1 inhibitors in the presence of 10 μM nocodazole or 1 μM Taxol together with 10 μM MG132 for 1 h, and then harvested. For preparation of microtubule-depolymerized fractions, cells were washed once with PBS, lysed with lysis buffer containing 1% NP-40 and incubated for 5 min on ice. After centrifugation at 16,000 × *g* for 5 min at 4 °C, pellet fractions were washed twice with lysis buffer and sonicated in sample buffer. The supernatants were centrifuged at 100,000 × *g* for 30 min at 4 °C, and supernatants were collected. Similarly, to prepare microtubule-polymerized fractions, cells washed with PEM buffer (100 mM PIPES pH 6.9, 1 mM EGTA, 1 mM MgCl_2_) including phosphatase and protease inhibitor cocktails were lysed in PEM buffer in the presence of 20 μM Taxol, 1% NP-40 and 1 mM GTP. After incubation at RT for 1 min, cell lysates were cleared by centrifugation at 16,000 × *g* for 5 min at RT, and then, pellet fractions were washed twice with lysis buffer and sonicated in sample buffer. After centrifugation at 100,000 × *g* for 30 min at 4 °C, supernatants were collected. Equivalent amounts of supernatant and pellet fractions were analysed by immunoblotting.

## Electronic supplementary material

Supplementary Figures
