# Supplementary material for: Plk1 bound to Bub1 contributes to spindle assembly checkpoint activity during mitosis
Source: Sci Rep. 2017 Aug 18;7:8794. doi: 10.1038/s41598-017-09114-3 (PMC5562746; doi:10.1038/s41598-017-09114-3)
Supplement: Supplementary file 1 — Supplementary Figures [file 41598_2017_9114_MOESM1_ESM.pdf]

Supplementary Information

Plk1 bound to Bub1 contributes to spindle assembly checkpoint activity during mitosis

Masanori Ikeda and Kozo Tanaka*

Department of Molecular Oncology, Institute of Development, Aging and Cancer, Tohoku University, 4-1 Seiryo-machi, Aoba-ku, Sendai, Miyagi 980-8575, Japan

Correspondence and requests for materials should be addressed to K.T. (E-mail: kozo.tanaka.d2@tohoku.ac.jp)
Tel and Fax: +81-22-717-849

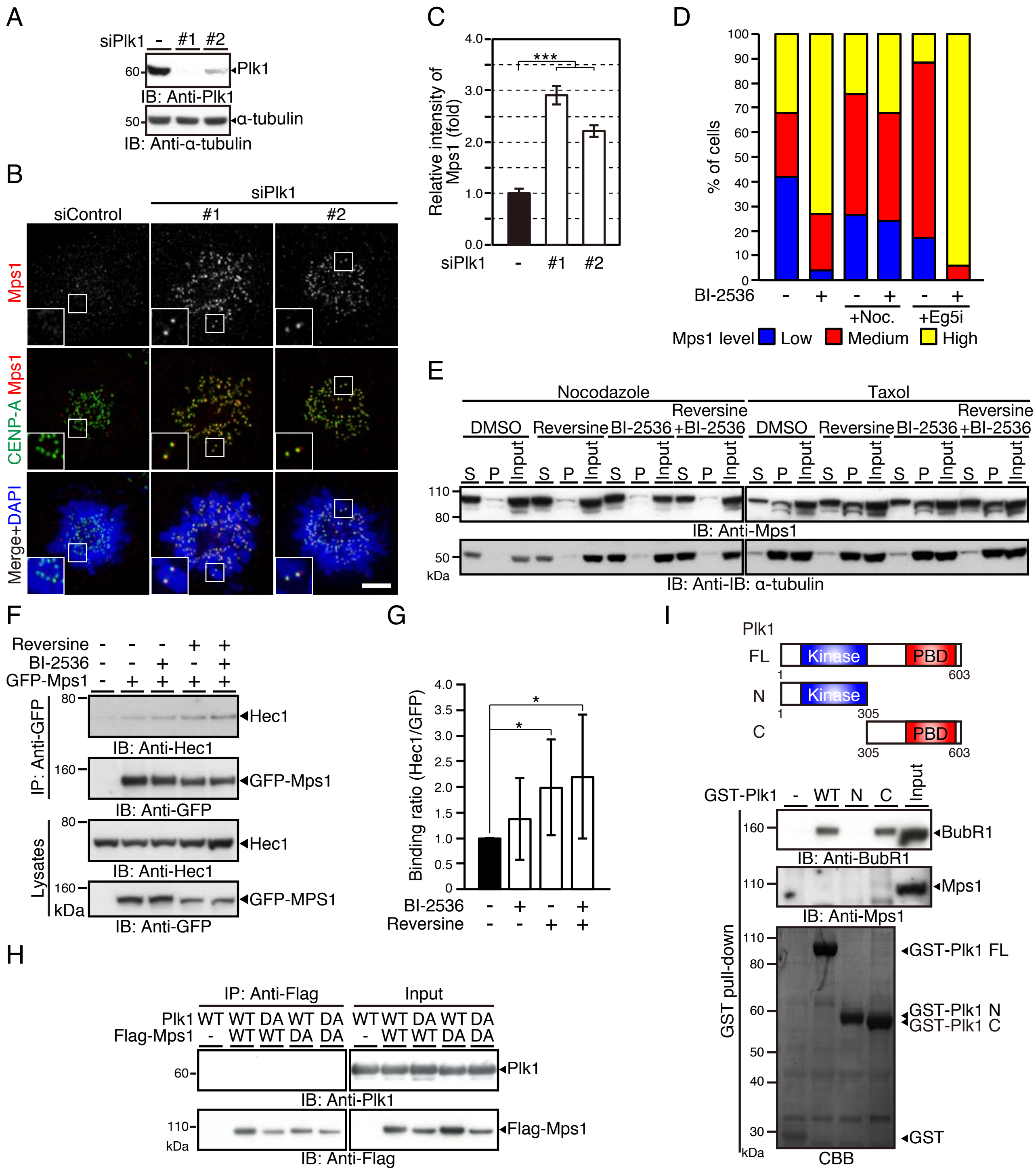

Supplementary Figure S1. Mps1 on kinetochores increases by downregulation of Plk1. (**A**) Efficiency of Plk1 depletion in HeLa cells. Lysates of cells transfected with two independent siRNAs against Plk1 were subjected to immunoblot analysis using an antibody against Plk1 or α-tubulin. (**B**) Mps1 on kinetochores increases in Plk1-depleted mitotic cells. Cells expressing CENP-A-GFP (green) were transfected with siRNAs against Plk1 and arrested in mitosis by inhibiting Eg5. These cells were fixed and immunostained with an antibody against Mps1 (red), and counterstained with DAPI for DNA (blue). Scale bar: 5 μm. Boxed areas are magnified in the lower left corner of the each panel. (**C**) Mps1 levels on kinetochores in cells depleted of Plk1 treated with Eg5 inhibitor III. At least 106 kinetochores per cell (5 cells) from a single experiment, representing three independent experiments, were counted for each condition. Signal intensity in control cells was set as 1. Error bars represent S.E. *** $P<0.0001$ (two-tailed *t*-test). (**D**) Mps1 level on kinetochores in cells treated with or without BI-2536 in the presence of nocodazole or Eg5 inhibitor III. Signal level of kinetochore Mps1 in each cell was classified in three categories (low, medium, and high). Fifty cells were counted for each condition. The experiments were repeated three times, and the representative data derived from a single experiment were shown. (**E**) Microtubule pelleting assay with Plk1- and/or Mps1-inhibited cell lysates. Mitotic HeLa cells selectively collected by shake-off procedure were treated with reversine and/or BI-2536 in the presence of

nocodazole or taxol together with MG132. Cell lysates were separated by centrifugation, and the supernatant (S) and pellet (P) fractions were analyzed by western blotting with an Mps1-antibody or anti-α-tubulin antibody. (**F**) Hec1 interaction with Mps1. GFP-Mps1 was expressed in HeLa cells in the presence or absence of 500 nM reversine and/or BI-2536, and Hec1 associated with GFP-Mps1 was detected in the immunoprecipitates using an anti-GFP antibody. (**G**) Quantification of the binding ratio of Hec1 to GFP-Mps1 in (F). The ratio in the absence of kinase inhibitors was set as 1. Error bars represent S.D. of seven independent experiments. *P<0.05 (two-tailed t-test). (**H**) Plk1 does not bind to Mps1. Flag-Mps1 and Plk1, either wild type (WT) or a kinase-dead mutant (DA), were expressed in Sf-21 cells, and the purified proteins were subjected to an immunoprecipitation with an anti-Flag antibody, followed by western blot analysis with an anti-Plk1 or anti-Flag antibody. (**I**) Mps1 does not bind to Plk1 via PBD. Schematic diagrams of Plk1 deletion mutants used in the GST pull-down assay are shown. The location of kinase domain and polo-box domain (PBD) are shown in blue and red, respectively. Mitotic HeLa cell lysates were subjected to a GST pull-down assay using GST-tagged full length or the N- or C-terminal fragments of Plk1. Precipitates were analyzed by western blotting analysis with antibodies against Mps1 or BubR1.

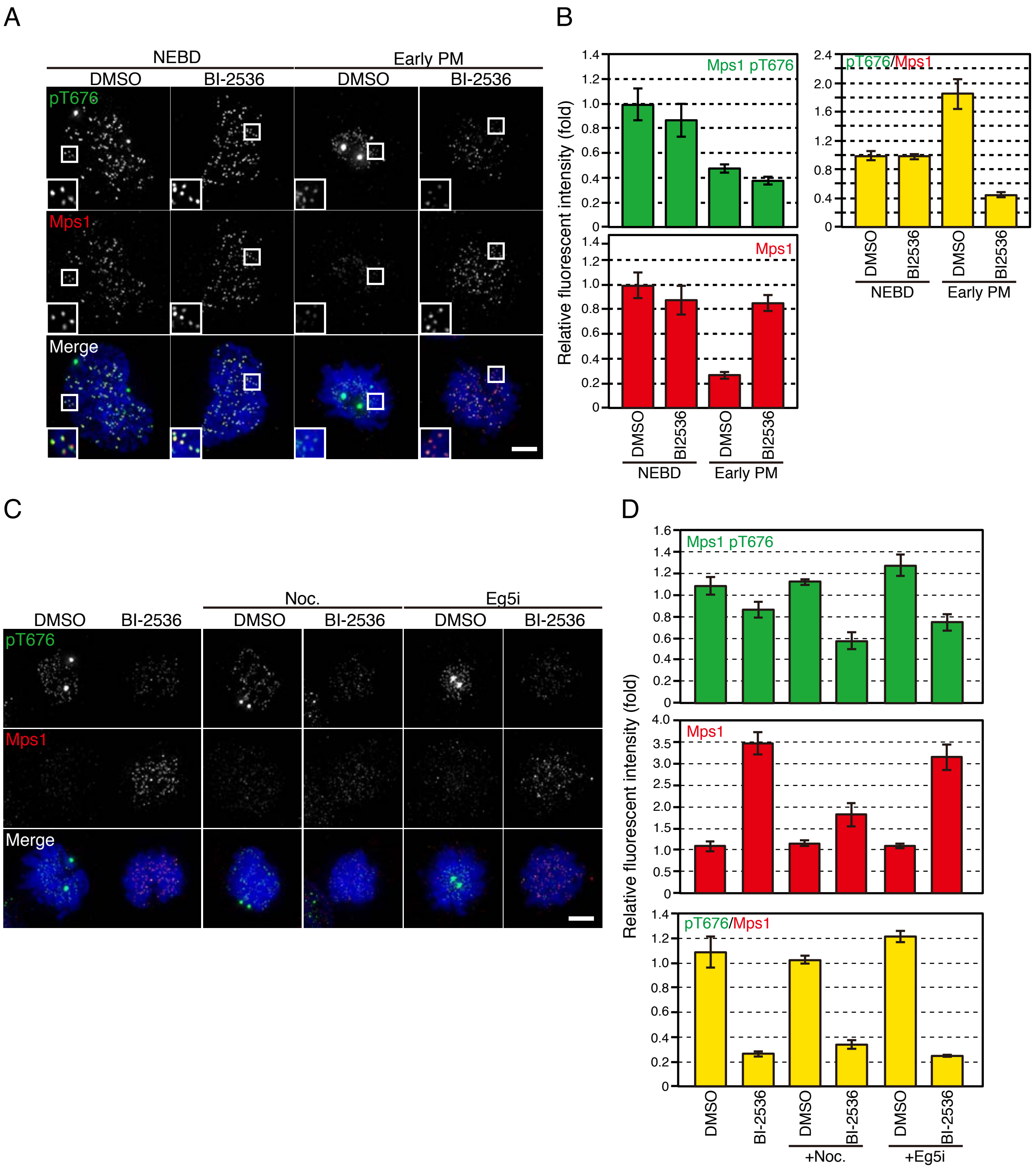

Supplementary Figure S2. Plk1 promotes phosphorylation of Mps1 on kinetochores. (**A**) Kinetochore localization of phosphorylated Mps1 in Plk1-inhibited cells. HeLa cells treated with or without BI-2536 were fixed and immunostained with an antibody against phosphorylated Mps1 at Thr676 (pT676; green) or total Mps1 (red). DNA was stained with DAPI (blue). Scale bar: 5 µm. Boxed areas are magnified in the lower left corner of the each panel. (**B**) Quantification of phosphorylated or total Mps1 on kinetochores. Fluorescence intensity of Mps1-pT676 (green), total Mps1 (red), and the ratio of Mps1-pT676 to total Mps1 (yellow) in cells treated as in (A) are shown. Signal intensity in DMSO-treated cells at NEBD was set as 1. More than 100 kinetochores per cell were quantified for each condition. Error bars represent S.E. The experiments were repeated three times, and the representative data obtained from a single experiment were shown. (**C**) Kinetochore localization of phosphorylated Mps1 in Plk1-inhibited cells in the presence of nocodazole or Eg5 inhibitor III. HeLa cells treated with DMSO, nocodazole, or Eg5 inhibitor III with or without BI-2536 were immunostained as in (A). DNA was stained with DAPI (blue). Scale bar: 5 µm. (**D**) Quantification of phosphorylated or total Mps1 on kinetochores in cells treated as in (C). Fluorescence intensity of Mps1-pT676 (green), total Mps1 (red), and the ratio of Mps1-pT676 to total Mps1 (yellow) obtained from a single experiment, representing three independent experiments, are shown as in (B). Signal intensity in DMSO-treated cells without nocodazole and Eg5 inhibitor III was set as 1. At least 75 kinetochores per cell were quantified for each condition. Error bars represent S.E.

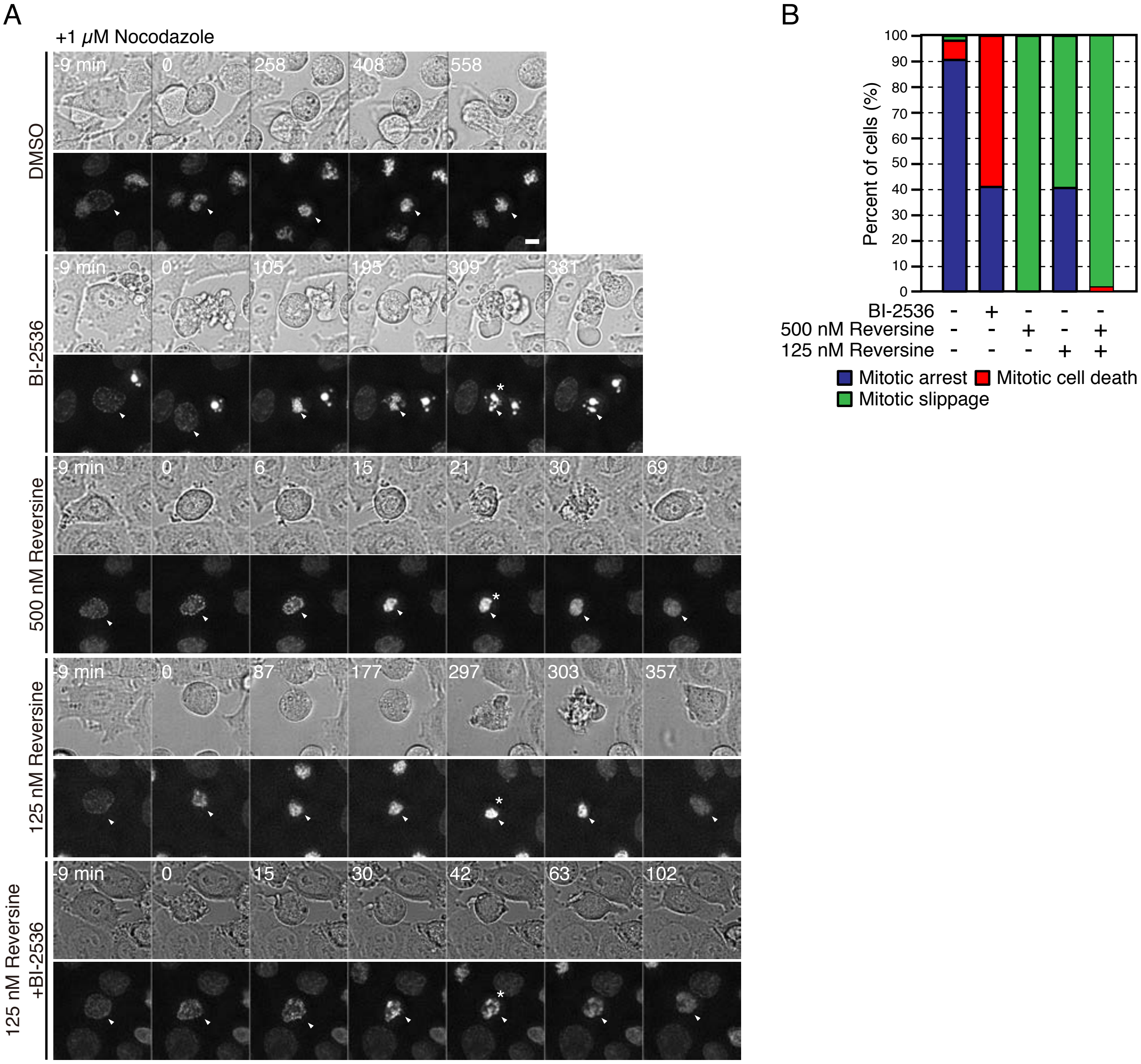

Supplementary Figure S3. Fate of mitotic HeLa cells treated with reversine- and/or BI-2536 (A) HeLa cells stably expressing H2B-mCherry treated with 1 μM nocodazole with or without reversine and/or BI-2536 were subjected to live cell imaging. Selected bright field and H2B-mCherry images were shown. Time is shown in minutes, and the time when the observed cell rounded up was set as 0 min. Arrowheads indicate H2B-mCherry signal of observed cells during the recording, and asterisks show the timing of mitotic exit, followed by mitotic cell death or mitotic slippage. Scale bar represents 10 μm. (**B**) Quantification of cells exhibiting mitotic arrest, mitotic cell death, or mitotic slippage. Percentage of cells treated as in (A) that showed each cell fate was shown. Data were derived from at least 43 cells per condition from a single experiment, representing three independent experiments.

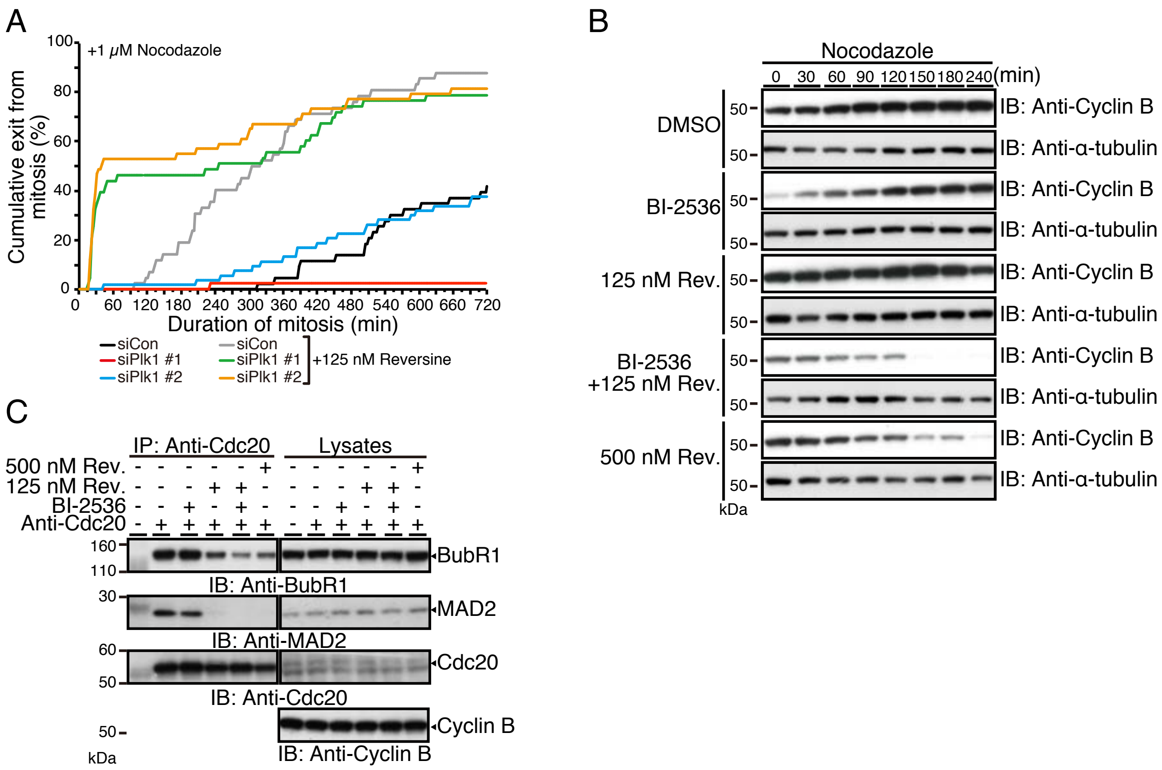

Supplementary Figure S4. Plk1 is involved in SAC maintenance. (**A**) A graph showing cumulative frequency of cells exited from mitosis in the presence of 1 µM nocodazole with or without 125 nM reversine in Plk1-depleted cells. At least 39 cells depleted of Plk1 were analyzed for each condition. The experiments were repeated three times, and the representative data obtained from a single experiment were shown. (**B**) Expression of Cyclin B in nocodazole-treated cells treated with reversine (Rev) and/or BI-2536. Total lysates of mitotic HeLa cells collected during the time course by shake-off procedure and treated with indicated kinase inhibitors together with 1 µM nocodazole were separated by SDS-PAGE and probed by western blotting with an anti-Cyclin B antibody or anti-α-tubulin antibody. (**C**) Formation of the mitotic checkpoint complex (MCC) in cells treated with reversine (Rev) and/or BI-2536. HeLa cells were incubated with reversine and/or BI-2536 in the presence of 1 µM nocodazole and 10 µM MG132. Lysates of mitotic cells collected as in (B) were subjected to immunoprecipitation (IP) with an anti-Cdc20 antibody. Total cell lysates and IPs were separated by SDS-PAGE and probed by western blotting with antibodies as indicated.

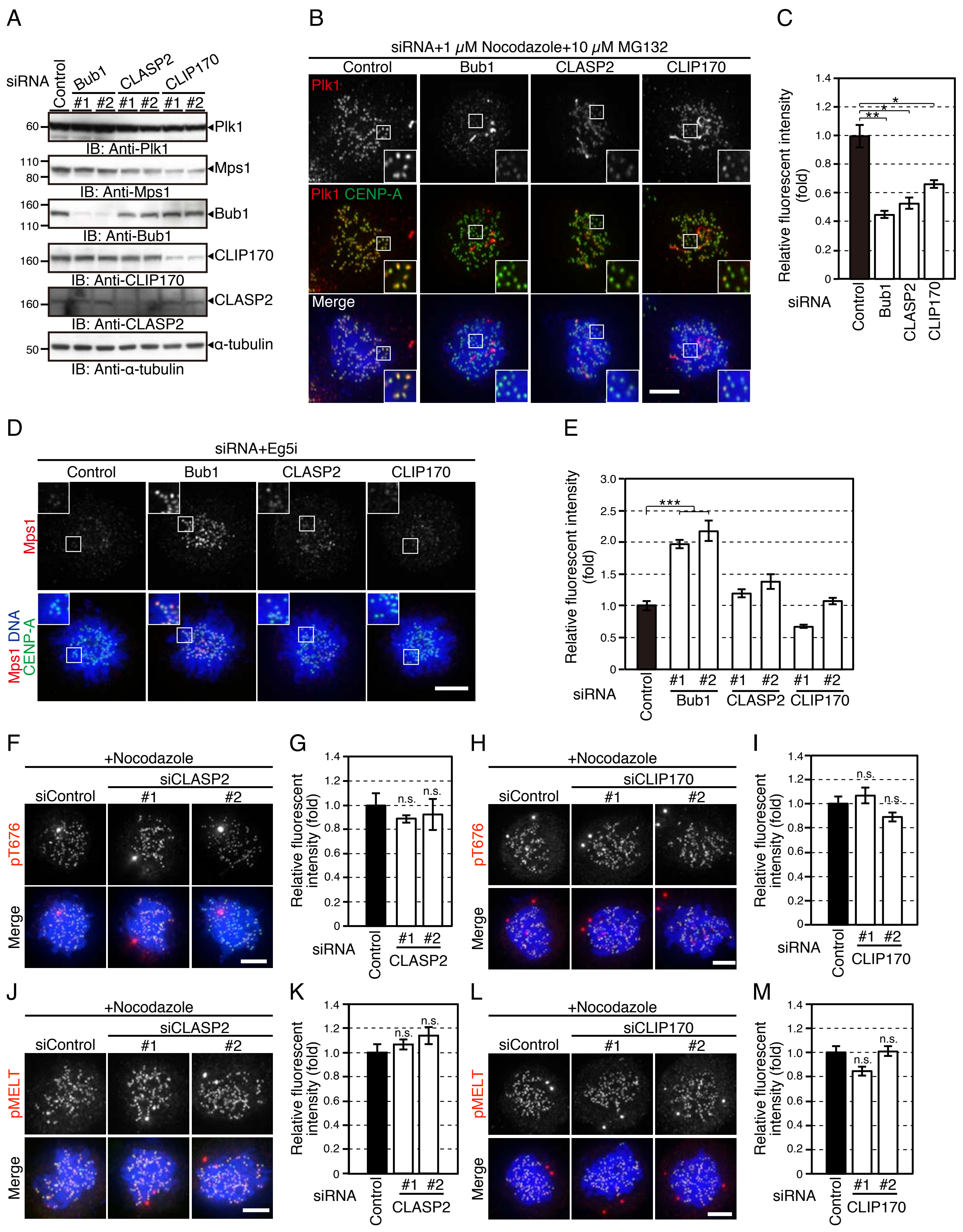

Supplementary Figure S5. Bub1, but not CLASP2 or CLIP-170, is involved in Mps1 and Knl1 phosphorylation. (**A**) Depletion of Bub1, CLASP2, and CLIP-170 with RNAi. Cells transfected with respective siRNAs were lysed and subjected to western blot analysis with antibodies as indicated. (**B**) Plk1 localization on kinetochores in cells depleted of Bub1, CLASP2, and CLIP-170. Cells expressing CENP-A-GFP (green) transfected with respective siRNAs were treated with nocodazole and MG132, and immunostained with antibodies against Plk1 (red). DNA was stained with DAPI (blue). Scale bar: 5 µm.

Boxed areas are magnified in the lower right corner of the each panel. (**C**) Quantification of Plk1 on kinetochores. Fluorescence intensity of Plk1 in cells treated as in (B) obtained from a single experiment, representing three independent experiments, is shown. Signal intensity in DMSO-treated cells was set as 1. At least 80 kinetochores per cell (5 cells) were measured per condition. Error bars represent S.E. **P*<0.05; ***P*<0.005 (two-tailed *t*-test). (**D**) Mps1 localization on kinetochores in cells depleted of Bub1, CLASP2, and CLIP-170. Cells expressing CENP-A-GFP (green) transfected with respective siRNAs were treated with Eg5 inhibitor III, and immunostained with an antibody against Mps1 (red). DNA was stained with DAPI (blue). Scale bar: 5 μm. Boxed areas are magnified in the upper left corner of the each panel. (**E**) Quantification of Mps1 on kinetochores. Fluorescence intensity of Mps1 in cells treated as in (D) is shown. Signal intensity in DMSO-treated cells was set as 1. At least 93 kinetochores per cell were quantified per condition. The experiments were repeated three times, and the representative data derived from a single experiment were shown. Error bars represent S.E. ****P*<0.0005 (two-tailed *t*-test). (**F-I**) Phosphorylation of Mps1 in CLASP2- (F) or CLIP-170- (H) depleted HeLa cells. Cells treated with nocodazole were fixed and staind with antibodies against phospho-Thr676 (pT676) of Mps1 (red). DNA was stained with DAPI (blue). Scale bar: 5 μm. Quantification of pT676 signal of Mps1 on kinetochores in (F) and (H) were shown in (G) and (I), respectively. At least 47 kinetochore signal per cell was measured for 5 cells per condition. The experiments were repeated three times, and the representative data obtained from a single experiment were shown. Fluorescence intensity in cells transfected with control siRNA was set as 1. n.s.; not significant (two-tailed *t*-test). (**J-M**) Phosphorylation of Knl1 at the MELT repeat in CLASP2- (J) or CLIP-170- (L) depleted HeLa cells. Cells expressing CENP-A-GFP (green) transfected with each siRNA were treated with nocodazle, fixed and stained with antibody against phosphorylated MELT repeat (pMELT; red). DNA was probed by DAPI (blue). Scale bar: 5 μm. Quantification of pMELT signal on kinetochores in (J) and (L) obtained from a single experiment, representing three independent experiments, were shown in (K) and (M), respectively. Fluorescence intensity of the pMELT was measured for at least 57 kinetochores per cell for 5 cells per condition. Signal intensity in cells transfected with control siRNA was set as 1. n.s.; not significant (two-tailed *t*-test).

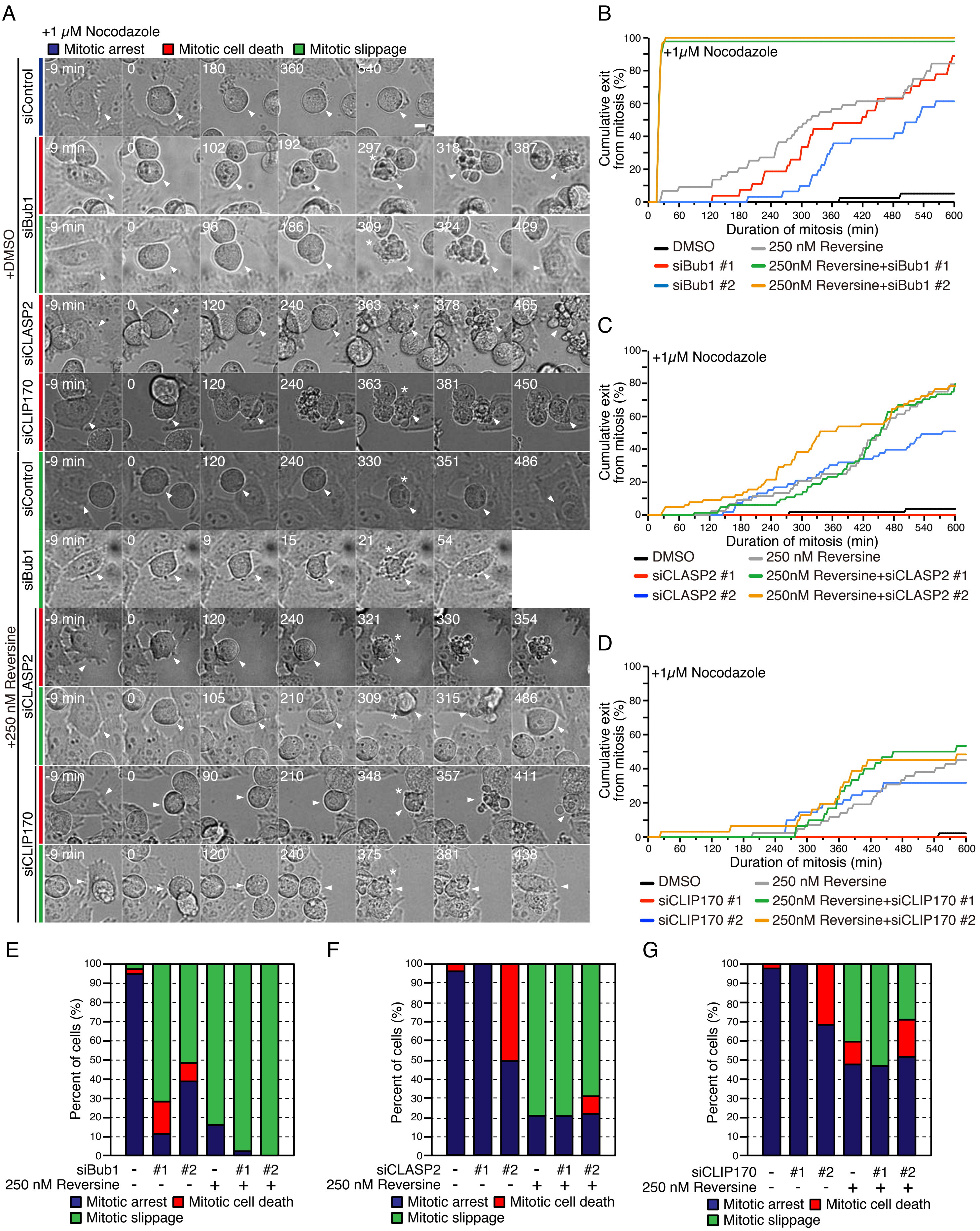

Supplementary Figure S6. Fate of mitotic HeLa cells depleted of Bub1, CLASP2 or CLIP-170. (A) A series of selected bright field images from a live imaging of HeLa cells depleted of Bub1, CLASP2 or CLIP-170 in the presence of 1 μM nocodazole with or without 250 nM reversine. Time is shown in

minutes, and the time when the observed cell rounded up was set as 0 min. Arrowheads indicate observed cells during the recording, and asterisks show the timing of mitotic exit, followed by mitotic cell death or mitotic slippage. Scale bar represents 10 μm. (**B-D**) Graphs showing cumulative frequency of cells exited from mitosis in the presence of 1 μM nocodazole and 250 nM reversine. Cells were transfected with or without siRNAs for Bub1 (B), CLASP2 (C), or CLIP-170 (D). At least 27 cells were quantified per condition. All of the graphs were obtained from a single experiment, representing three independent experiments. (**E-G**) Quantification of cells exhibiting mitotic arrest, mitotic cell death or mitotic slippage. Percentage of cells treated as in (A) that showed each cell fate was shown. Data obtained from a single experiment, representing three independent experiments, were derived from at least 27 cells per condition.

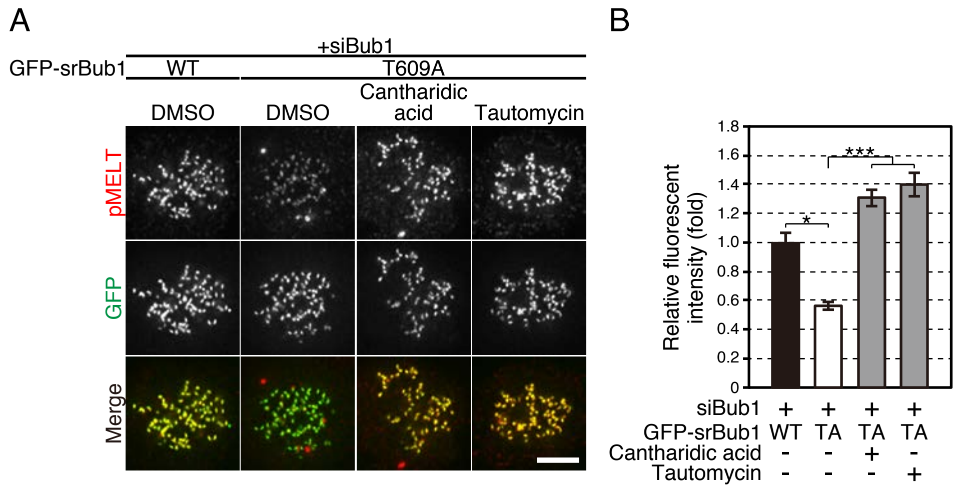

Supplementary Figure S7. Plk1 bound to Bub1 counteracts phosphatase activity for Knl1 phosphorylation. (**A**) Knl1 phosphorylation at the MELT repeats in Bub1-depleted cells expressing siRNA-resistance Bub1 constructs in the presence of phosphatase inhibitors. Cells treated as in Fig. 3B, and cells expressing Bub1-T609A were exposed to 50 μM cantharidic acid or 3 μM tautomycin before fixation together with 10 μM MG132. Cells were stained with an antibody against a phosphorylated MELT repeat (pMELT; red) or GFP (green). Scale bar: 5 μm. (**B**) Quantification of Knl1-pMELT signal on kinetochores. Signal intensity of Knl1-pMELT in cells treated as in (A) was obtained from a single experiment, representing three independent experiments, and measured from at least 41 kinetochores per cell (5 cells) per condition. Error bars represent S.E. *$P<0.01$; ***$P<0.0005$ (two-tailed t-test).

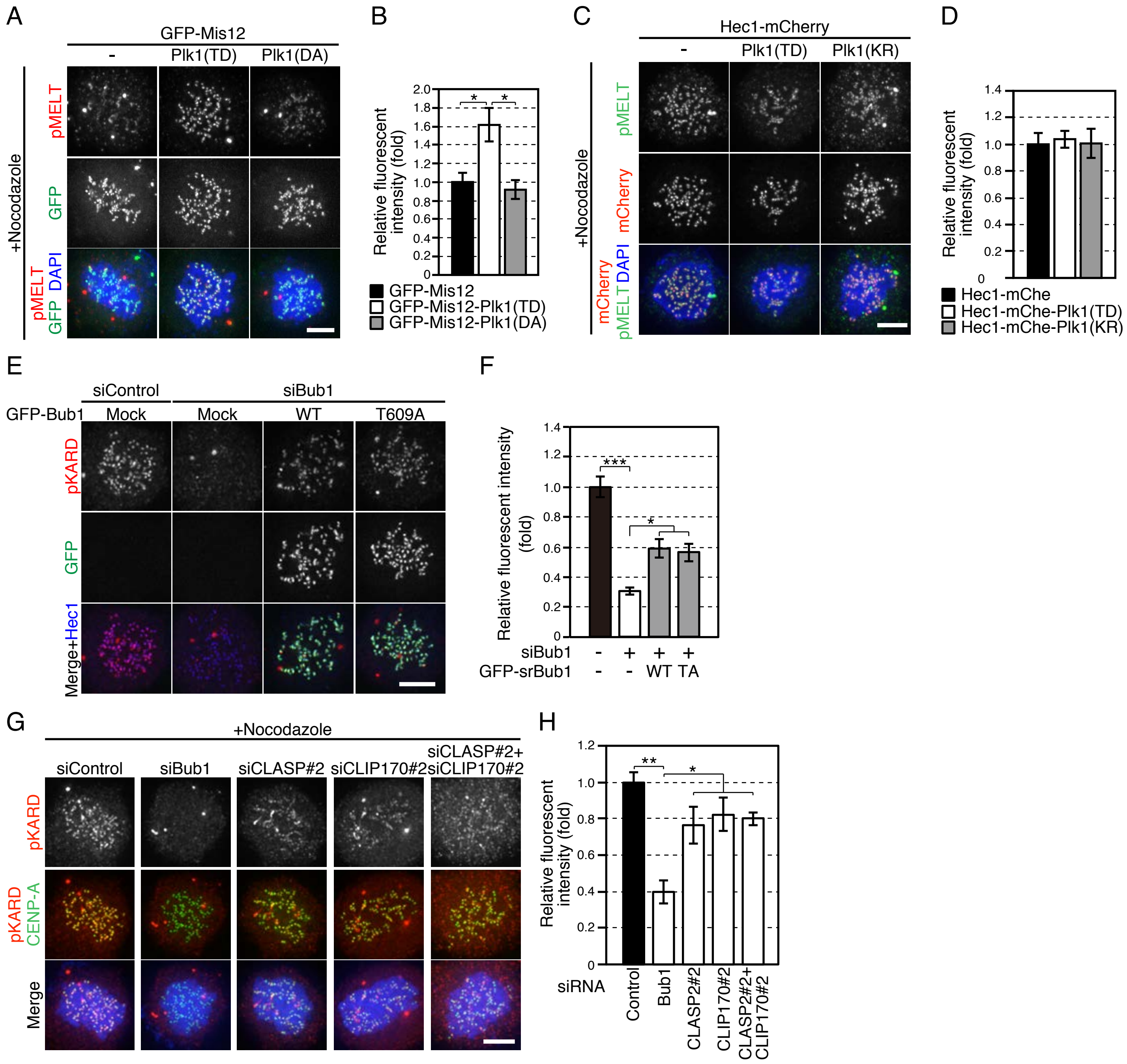

Supplementary Figure S8. Plk1 tethered to Mis12, not to Hec1, enhances Knl1 phosphorylation. (**A**) Phosphorylation of Knl1 at the MELT repeat in cells ectopically expressing GFP-Mis12-Plk1. Cells transfected with GFP-Mis12 or GFP-Mis12-Plk1 construct (constitutively active form (T210D; TD) or kinase-dead form (D194A; DA)) were treated with nocodazole, fixed and stained with antibodies against phosphorylatable MELT repeat (pMELT; red) and GFP (green). DNA was stained with DAPI (blue). Scale bar: 5 µm. (**B**) Quantification of pMELT signal in cells shown in (A). At least 40 kinetochore signal of pMELT was measured per cell (5 cells) per condition. Error bars represent S.E. *P<0.05 (two-tailed t-test). (**C**) Knl1 phosphorylation at the MELT repeats in cells expressing Hec1-mCherry-Plk1 (Hec1-mChe-Plk1). HeLa cells transfected with Hec1-mChe or Hec1-mChe-Plk1 construct (constitutively-active form (T210D; TD) or kinase-inactive form (K82R; KR)) were treated as in (A), fixed and stained with antibody against phosphorylatable MELT repeat (pMELT; green) and mCherry (red). DNA was probed by DAPI (blue). Scale bar: 5 µm. (**D**) Quantification of pMELT signal in Knl1 on kinetochores. Fluorescence intensity of pMELT in cells treated as in (C) was quantified for at least 46 kinetochores per cell (5 cells) per condition. Error bar represent S.E. *P<0.05 (two-tailed t-test).

(**E**) BubR1 phosphorylation at the KARD domain in Bub1-depleted cells. Cells were treated as in Fig. 3B and stained with an antibody against phosphorylated KARD domain (pKARD; red), GFP (green), or Hec1 (blue). Scale bar: 5 μm. (**F**) Quantification of BubR1-pKARD signal on kinetochores. Fluorescence intensity of BubR1-pKARD in cells treated as in (E) was measured for at least 44 kinetochores per cell for 5 cells per condition. Error bars represent S.E.*P<0.05; ***P<0.0005 (two-tailed *t*-test). (**G**) BubR1 phosphorylation at the KARD domain in Bub1-, CLASP2-, CLIP-170-, or CLASP2/CLIP-170-depleted cells. HeLa cells transfected with indicated siRNAs were arrested in mitosis with nocodazole, fixed and stained with antibodies against phosphorylatable KARD domain of BubR1 (pKARD; red) and GFP (green). DNA was stained with DAPI (blue). Scale bar: 5 μm. (**H**) Quantification of pKARD signal on kinetochores. Signal intensity of pKARD in cells treated as in (G) was obtained from at least 73 kinetochores per cell for 5 cells per condition. Error bars represent S.E. *P<0.05; ** P<0.005 (two-tailed *t*-test). All of the quantitative analysis were obtained from a single experiment, representing three independent experiments.

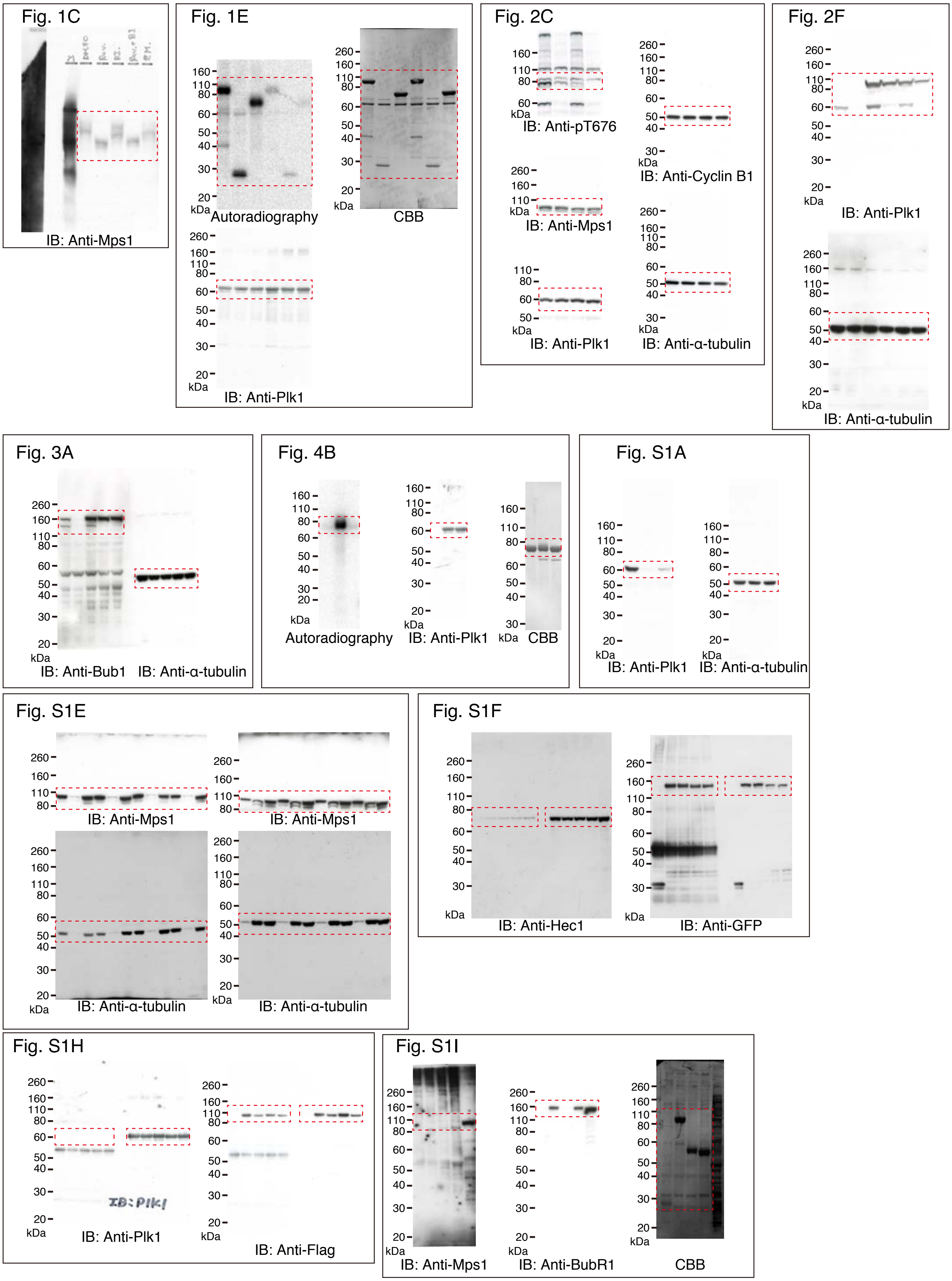

Supplementary Figure S9. Uncropped images of immunoblots and membranes.

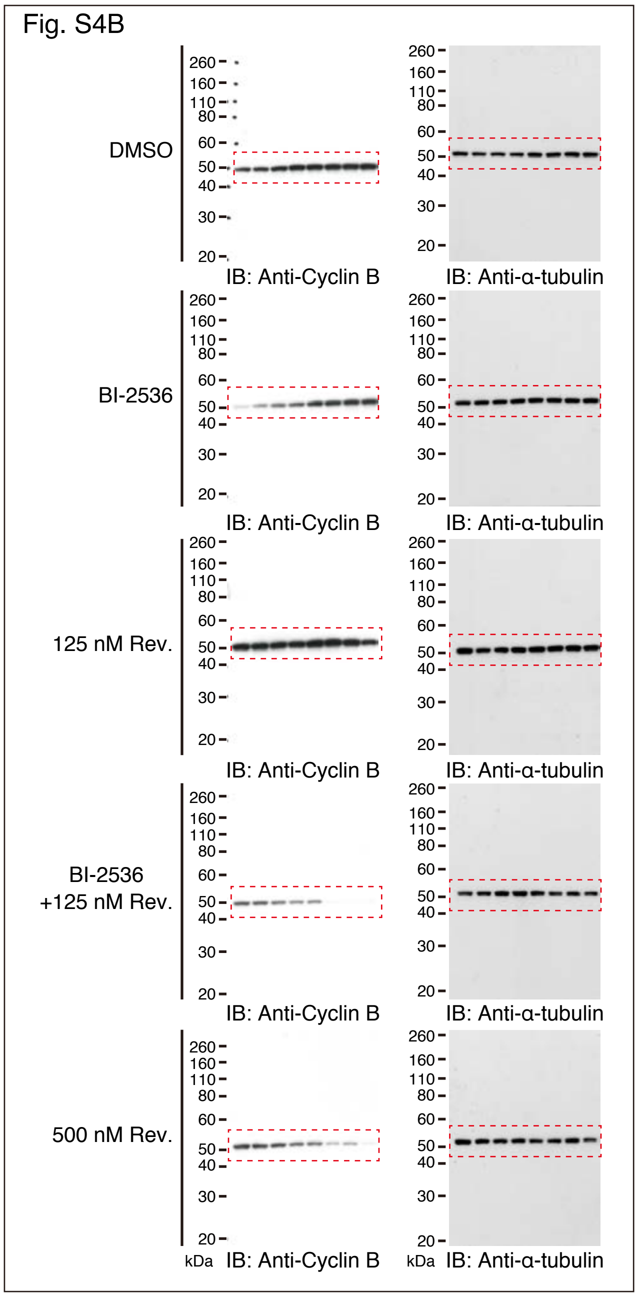

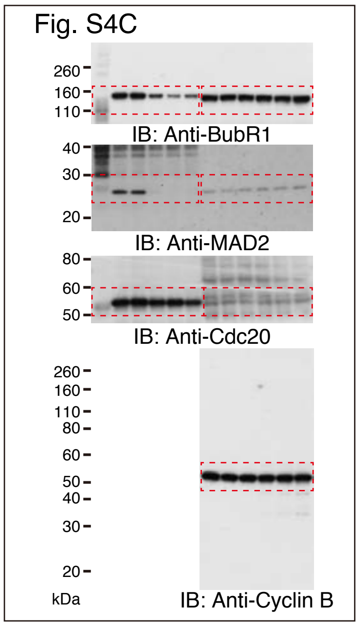

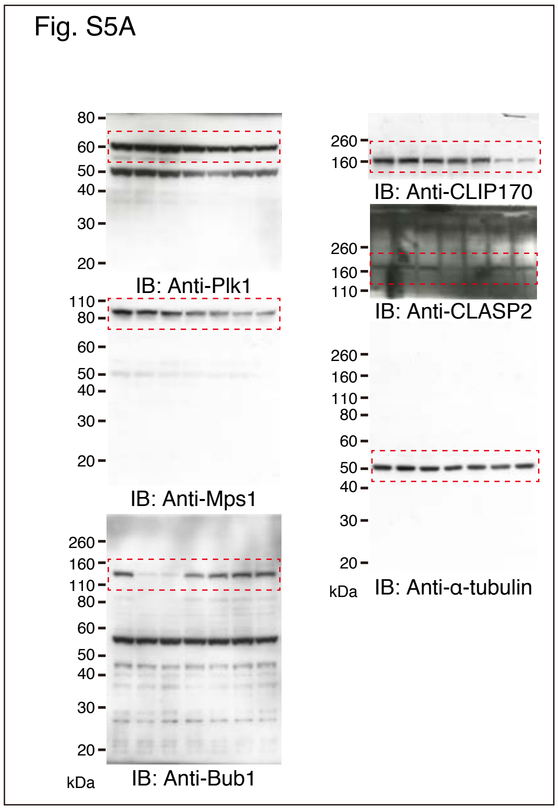

Supplementary Figure S9. continued
